# A Comparative Perspective on Functionally-Related, Intracellular Calcium Channels: The Insect Ryanodine and Inositol 1,4,5-Trisphosphate Receptors

**DOI:** 10.3390/biom11071031

**Published:** 2021-07-15

**Authors:** Umut Toprak, Cansu Doğan, Dwayne Hegedus

**Affiliations:** 1 Molecular Entomology Laboratory, Department of Plant Protection, Faculty of Agriculture, Ankara University, Ankara 06110, Turkey; 7cansudogan@gmail.com; 2 Agriculture and Agri-Food Canada, Saskatoon, SK S7N 0X2, Canada; dwayne.hegedus@canada.ca; 3 Department of Food and Bioproduct Sciences, College of Agriculture and Bioresources, University of Saskatchewan, Saskatoon, SK S7N 5A8, Canada

**Keywords:** ryanodine receptor, inositol 1,4,5-trisphosphate receptor, calcium channel, endoplasmic reticulum, pest control, diamide

## Abstract

Calcium (Ca^2+^) homeostasis is vital for insect development and metabolism, and the endoplasmic reticulum (ER) is a major intracellular reservoir for Ca^2+^. The inositol 1,4,5- triphosphate receptor (IP_3_R) and ryanodine receptor (RyR) are large homotetrameric channels associated with the ER and serve as two major actors in ER-derived Ca^2+^ supply. Most of the knowledge on these receptors derives from mammalian systems that possess three genes for each receptor. These studies have inspired work on synonymous receptors in insects, which encode a single IP_3_R and RyR. In the current review, we focus on a fundamental, common question: “why do insect cells possess two Ca^2+^ channel receptors in the ER?”. Through a comparative approach, this review covers the discovery of RyRs and IP_3_Rs, examines their structures/functions, the pathways that they interact with, and their potential as target sites in pest control. Although insects RyRs and IP_3_Rs share structural similarities, they are phylogenetically distinct, have their own structural organization, regulatory mechanisms, and expression patterns, which explains their functional distinction. Nevertheless, both have great potential as target sites in pest control, with RyRs currently being targeted by commercial insecticide, the diamides.

## 1. Introduction

Calcium (Ca^2+^) is a key second messenger that plays important roles in numerous cellular and physiological processes, including cell motility, membrane transport processes, gene expression and regulation, nuclear pore regulation, vesicle fusion, neurotransmission, muscle contraction, hormone biosynthesis, and apoptosis [1]. Similar to other animals, Ca^2+^ is also essential for insects [2] where it is involved in development and metamorphosis [3], reproduction [4], sex pheromone synthesis [5], cold sensing [6], neurotransmitter release [7], olfactory responses [8], carbohydrate [9] and lipid metabolism [10], and diapause [11]. Due to these essential roles, it is critical to maintain cellular Ca^2+^ homeostasis [12].

In animal cells, Ca^2+^ homeostasis is coordinated through channels, transporters and pumps located in the plasma membrane, the endoplasmic reticulum (ER) [13], as well as other organelles, such as the Golgi apparatus [14], mitochondria [15], and lysosomes [16]. Calcium binding proteins in the cytosol or organelles are also involved in the maintenance of Ca^2+^ levels by functioning as calcium buffers [10,11]. Extracellular Ca^2+^ concentrations are relatively high (1–2 mM), while the cytoplasm of most cells contains much lower resting Ca^2+^ concentrations (in the 100 nM range) [17]. Calcium entry via the plasma membrane is a major route to supply Ca^2+^ needed for the cell; however, cellular organelles, in particular the ER (sarcoplasmic reticulum—SR for muscle cells) (100–500 μM), supply Ca^2+^ and trigger Ca^2+^ signals rapidly when the intracellular levels of Ca^2+^ are low [17]. This occurs through the activation of intracellular Ca^2+^ channels associated with the ER. The two major Ca^2+^ release channels are the inositol 1,4,5-trisphosphate receptor (IP_3_R), activated by the secondary messenger inositol 1,4,5-trisphosphate (IP_3_), Ca^2+^, and the ryanodine receptor (RyR), named after its high affinity for the plant alkaloid ryanodine, which is mainly activated by Ca^2+^ and possibly by other secondary messengers [18,19,20,21,22]. The IP_3_R and RyR are both members of a family of tetrameric intracellular Ca^2+^-release channels and are encoded by single genes in insects, whereas humans possess three IP_3_R (IP_3_R1–3) and RyR (RyR1–3) genes with distinct tissue expression patterns and subcellular localization. Both receptors activate Ca^2+^ release from the ER/SR to the cytosol or other organelles; therefore, they serve as major links between extra- and intracellular stimuli, leading to regulation of various cellular processes [13,21]. It is noteworthy that they can also be associated with mitochondria [23,24,25] or membrane contact sites [26,27]. 

It is an ongoing question as to why animals possess two similar biochemical tools (RyR and IP_3_R) associated with the ER for the coordination of intracellular Ca^2+^ homeostasis [28]. Studies on the structure and localization of these channels together with expression, mutation, recombination, and functional genomic studies have provided important clues in distinguishing the functional attributes of RyR or IP_3_R channels in mammalian models. The two receptors also share structural and functional features in insects. Studies on insect IP_3_Rs and RyRs have been limited but have increased significantly in the last decade. Cloning of the genes encoding these receptors together with structural and functional analyses have provided important insights into our understanding of the role of these receptors in intracellular Ca^2+^ homeostasis, lipid metabolism, muscle function, neuronal signaling in relation to photoreceptors, olfaction, locomotor activities, and development in insects. The discovery of the diamide group of insecticides, which selectively target insect RyRs and affect Ca^2+^ homeostasis, has focused attention on these receptors and IP_3_Rs. In the current review, we first introduce the RyRs and IP_3_Rs from mammalian models that inspired the discovery of their insect counterparts (Section 2). We then present insect IP_3_Rs and RyRs from a comparative perspective according to their structure (Section 3), their involvement in the Ca^2+^ metabolic pathways (Section 4), functions (Section 5), and their potential as targets in pest control (Section 6).

## 2. Discovery of RyRs and IP_3_Rs

The first RyR gene (RyR1) was first isolated from rabbit skeletal muscle [29], followed by isolation of the rabbit cardiac muscle isoform (RyR2) [30]. A third isoform (RyR3), distinct from both the skeletal and cardiac muscle isoforms, was isolated from rabbit brain [31]. In contrast to mammals, insect genomes encode only one RyR. The first insect RyR was identified from *Drosophila melanogaster* (Diptera: Drosophilidae) [32,33]. The *D*. *melanogaster* RyR shows approximately 45%–47% amino acid identity with the three mammalian RyRs. RyRs have since been identified from the lepidopterans *Heliothis virescens* (Noctuidae) [34,35], *Bombyx mori* (Bombycidae) [36], *Cnaphalocrocis medinalis* (Crambidae) [37], *Plutella xylostella* (Plutellidae) [38,39], *Ostrinia furnacalis* (Crambidae) [40], *Helicoverpa armigera* (Noctuidae) [41], *Pieris rapae* (Pieridae) [42], *Chilo suppressalis* (Crambidae) [43,44], *Spodoptera exigua* (Noctuidae) [45], *Grapholita molesta* (Tortricidae) [46], *Tuta absoluta* (Gelechiidae) [47], and *S*. *frugiperda* [48], the dipteran *Bactrocera dorsalis* (Tephritidae) [49], the coleopterans *Tribolium castaneum* (Tenebrionidae) [50] and *Leptinotarsa decemlineata* (Chrysomelidae) [51], and the hemipterans *Laodelphax striatellus* (Delphacidae) [43], *Bemisia tabaci* (Aleyrodidae) [43], *Nilaparvata lugens* (Delphacidae) [52], *Sogatella furcifera* (Delphacidae) [53], *Myzus persicae* (Aphididae) [54], *Toxoptera citricida* (Aphididae) [55], *Dialeurodes citri* (Aleyrodidae) [56] (Table 1). 

The IP_3_R was first purified from rat cerebellum [57] and the gene encoding the first isoform (IP_3_R1) cloned from mouse cerebellum tissues [58]. This was followed by cloning of the IP_3_R2 isoform from rat brain [59] and IP_3_R3 from a rat insulinoma cell line [60]. Not surprisingly, the first insect IP_3_R was also identified from *D. melanogaster* [32,61]. The *D*. *melanogaster* IP_3_R has approximately 60% amino acid identity with the three mammalian IP_3_Rs, indicating a closer relatedness between mammalian and insect IP_3_Rs than to RyRs [32,61]. Compared to insect RyRs, an only limited number of studies on the identification of insect IP_3_Rs are available. IP_3_Rs have been identified from the coleopterans *T. castaneum* [50] and *L. decemlineata* [Doğan and Toprak, unpublished], from the hemipterans *B. tabaci* [62] and *M. persicae* [63] and the hymeopteran *Bombus terrestris* (Apidae) [63] (Table 1). 

**Table 1:** Insect ryanodine receptors (RyRs) and inositol triphosphate receptors (IP_3_Rs) identified to date.

Receptor	Species	Amino Acid (residue)	cDNA Size (bp)	Molecular Weight (kDa)	Reference
**RyRs**	**Lepidoptera**				
	*Bombyx mori* (Bombycidae)	5084	15,255 *	575	[36]
	*Cnaphalocrocis medinalis* (Crambidae)	5087	15,773	574	[37]
	*Plutella xylostella* (Plutellidae)	5123	15,748	579	[38]
		5164	16,113	584	[39]
	*Ostrinia furnacalis* (Crambidae)	5108	16,211	577	[40]
	*Helicoverpa armigera* (Noctuidae)	5142	16,083	581	[41]
	*Pieris rapae* (Pieridae)	5107	15,540	578	[42]
	*Chilo suppressalis* (Crambidae)	5133	16,392	581	[43]
		5133	16,102	581	[44]
		5128	15,402	580	[64]
	*Spodoptera exigua* (Noctuidae)	5118	15,748	579	[45]
	*Grapholita molesta* (Tortricidae)	5133	16,299	580	[46]
	*Tuta absoluta* (Gelechiidae)	5121	16,431	579	[47]
	*Spodoptera frugiperda*	5109	15,330	578	[48]
	**Diptera**				
	*Drosophila melanogaster* (Drosophilidae)	5134	15,405 *	581	[65]
	*Bactrocera dorsalis* (Tephritidae)	5140	15,750	582	[49]
	**Coleoptera**				
	*Tribolium castaneum* (Tenebrionidae)	5094	15,308	577	[50]
	*Leptinotarsa decemlineata* (Chrysomelidae)	5128	15,792	582	[51]
	**Hemiptera**				
	*Laodelphax striatellus* (Delphacidae)	5115	15,910	579	[43]
	*Bemisia tabaci* (Aleyrodidae)	5139	15,763	581	[43]
	*Nilaparvata lugens* (Delphacidae)	5140	15,735	581	[52]
	*Sogatella furcifera* (Delphacidae)	5128	15,985	579	[53]
	*Myzus persicae* (Aphididae)	5101	15,306 *	580	[54]
	*Toxoptera citricida* (Aphididae)	5101	15,639	580	[55]
	*Dialeurodes citri* (Aleyrodidae)	5126	15,538	579	[56]
**IP_3_Rs**	**Diptera**				
	*Drosophila melanogaster* (Drosophilidae)	2833	9558	319	[61]
	**Coleoptera**				
	*Tribolium castaneum* (Tenebrionidae)	2724	8175 *	309	[50]
	*Leptinotarsa decemlineata* (Chrysomelidae)	2736	8211 *	312	Doğan and Toprak, unpublished
	**Hemiptera**				
	*Bemisia tabaci* (Aleyrodidae)	2733	8202 *	311	[62]
	*Myzus persicae* (Aphididae)	3790	11,373 *	-	[63]
	**Hymenoptera**				
	*Bombus terrestris* (Apidae)	2727	10,966	309	[63]

* Translated region.

## 3. Structure of RyRs and IP_3_Rs

Both RyRs and IP_3_Rs are members of the voltage-sensitive ion channel (VIC) superfamily and form homomeric tetramers resembling a square mushroom. In mammalian RyRs, each monomer (~5000 amino acids) has a molecular weight of around 550–580 kDa, while each IP_3_R monomer (~2700 amino acids) has a molecular weight of around 260 kDa [22,66,67]. Several high-resolution structures of mammalian RyR [68,69,70,71,72,73] and IP_3_R domains [28,74,75,76,77,78] have been determined by X-ray crystallography, NMR, and cryogenic electron microscopy. RyRs and IP_3_Rs share 30–35% homology at the amino acid level and primarily consist of a large, N-terminal, hydrophilic domain (a.k.a. the “foot structure”), a dissimilar central modulatory domain, and a small, conserved, C-terminal domain with 6 transmembrane regions forming the Ca^2+^ conducting channel pore [73,79,80] (Table 2). Notably, the large N-terminal hydrophilic domain and the small C-terminal hydrophilic domains both face the cytoplasm. The N-terminal domain of IP_3_R forms the binding pocket for the native ligand IP_3_ and includes three subdomains, the IP_3_-binding core β (IBC-β) and α (IBC-α) which interact with IP_3_, and the suppressor (inhibitory) domain (SD) which reduces the affinity for IP_3_ [81,82,83,84,85]. Notably, IP_3_Rs without an SD bind IP_3_ with high affinity, but do not release Ca^2+^, suggesting the SD is essential for IP_3_-induced channel gating [82,84,86]. RyRs, although N-terminal domain does not bind IP_3_, have a similar arrangement as the N-terminal domain of IP_3_R and includes three subdomains termed A, B and C corresponding to the SD, IBC-β and IBC-α, respectively [28,87]. These lead to modulation of the gating of the Ca^2+^ pore that occurs between the fifth and sixth transmembrane segments in the carboxy-terminal domain [81,88]. The structural domains common to both RyRs and IP_3_Rs in mammalians are the **MIR** (**M**annosyltransferase, **I**P_3_R and **R**yR, pfam02815), **RIH** (**R**yR and **I**P_3_R **H**omology, pfam01365), and **RIH-associated** (pfam08454) domains [89] (Table 2). However, repeats termed the “**SPRY** domain (pfam00622)”, originally identified from *Dictyostelium discoideum* tyrosine kinase **sp**ore lysis A and the mammalian **Ry**Rs, and the “RyR domain (pfam02026)” are unique to RyRs [71,90,91,92]. The MIR domain is proposed to have a ligand transferase function [93], while the RIH domain might form the IP_3_ binding site together with the MIR domain in IP_3_Rs [94]. On the other hand, SPRY domains are typically known to mediate protein-protein interactions [95,96], while the function of RyR domain is unknown. The ryanodine-binding site is also localized to the carboxy terminus of both proteins within or close to the pore region [97]. Notably, the primary Ca^2+^ binding protein, calmodulin, interacts with RyRs in lipid bilayers [98] and binds to the RyR channel cytoplasmic assembly around 10 nm from the putative entrance to the transmembrane pore [99,100,101]. The N-terminal ligand-binding region of IP_3_R1 contains a calmodulin-binding domain that binds calmodulin independently of Ca^2+^ and mediates the inhibition of IP_3_ binding to IP_3_R1 [102].

**Figure 1:**
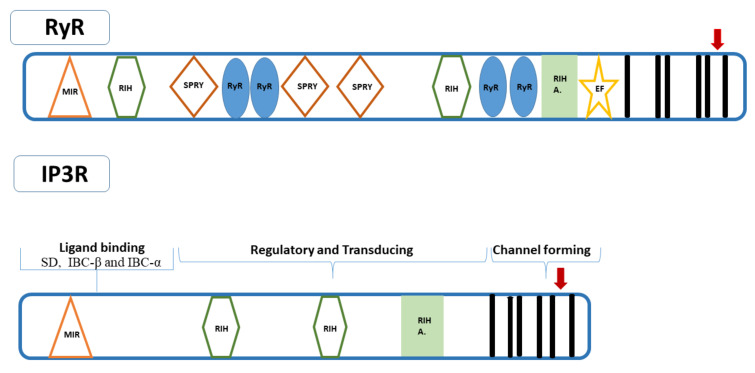
The conserved domains for RyR are listed as following MIR (Mannosyltransferase, IP_3_R, and RyR, pfam02815), RIH (RyR and IP_3_R Homology, pfam01365), the SPRY (spIA and RyR domains, pfam00622), RyR domain (pfam02026) [71,90,91,92], RIH A domains (RIH-associated, pfam08454) [89], EF-hands, and putative transmembrane domain (TM1-TM6). IP_3_R has three putative functional regions: ligand binding, central regulatory, and channel forming sites. Ligand binding region includes three subdomains, the IP_3_-binding core β (IBC-β) and α (IBC-α) that interact with IP_3_; and the suppressor domain (SD) reducing the affinity for IP_3_ [81,82,83,84,85]. The conserved domains for IP_3_R are listed as following MIR RIH, RIH A, and TM1-TM6. Arrow corresponding to TM5 and TM6 including the suppressor domain and ligand binding, which leads to modulation of the gating of the Ca^2+^ pore in both channels.

Insect RyRs are commonly composed of 5084–5164 residues with a molecular weight of 574–582 kDa. Crystal structures of the *P. xylostella* RyR N-terminal domain [103], Repeat34 domain [104] and SPRY2 domain [105], and the N-terminal domain of *Apis mellifera* RyR [106] are the only ones available. Therefore, the entire structural domain organization and key regions of insect RyRs are based on limited X-ray crystallography predictions and comparative modeling studies using the mammalian counterparts [107]. These studies revealed that the basic structure of insect RyRs is similar to their mammalian counterparts (Table 2). Insect RyRs are commonly composed of a large amino-terminal region including a MIR domain, two RIH domains, three SPRY domains, four RyR repeat domains, one RIH-associated domain, and a carboxy-terminal region including six transmembrane domains and two calcium-binding EF-hand domains [49,50,53,55,56] (Figure 1). Recently, Lin et al. [107] generated multiple structural models of *P*. *xylostella* RyR based on the rabbit RyR1 cryo-EM structure. This revealed that PxRyR is highly modular and consists of 20 individual domains, including 3 N-terminal domains, 3 SPRY domains, 3 insect divergent regions (IDR), 2 RYR repeat domains, 3 solenoid [SOL] domains, a shell-core linker peptide (SCLP) domain, an EF-hand domain (EF1&2), a thumb and forefinger (TaF) domain, a pseudo voltage-sensor domain (pVSD), a pore-forming (PF) domain and a C-terminal domain (CTD) with six transmembrane helices. There is evidence indicating the N-terminal cytoplasmic domain modulates the gating of the channel pore located in the C-terminus similar to that in mammalian RyRs [49,53,56,103,106]. The proposed pore (loop), including the characteristic “GXRXGGGXGD” motif [108], is located between the C-terminal helices 5 and 6 [37,39,41,109]. Notably, the loop is proposed to act as a selectivity filter for ions in both mammalian RyRs and IP_3_Rs, suggesting it also likely to enable the channels to discriminate between ions in insects. It is also worth noting that mutagenesis of residues in this region of both RyR and IP_3_R impairs channel conductance in mammalians [108,110,111]. Residues I^5023^, R^5039^, and D^5043^ (numbering based on *P. xylostella* RyR- GenBank accession number AET09964) [39] between TM5 and TM6 are conserved in insect RyRs [46,49,50,55,56] and the corresponding residues (I^4897^, R^4913^, and D^4917^) in rabbit RyR1 play role in the activity and conductance of the Ca^2+^ release channel [30,112]. A glutamate residue proposed to be involved in Ca^2+^ sensitivity in rabbit RyR1 (E^4032^) [113] and RyR3 (E^3885^) [114] is also conserved in insect RyRs (E^4201^ in PxRyR) [46,50]. The lepidopteran RyRs show sequence divergence from other insect RyRs in the carboxy-terminal region, especially in the region proximal to the pore-forming segment [37]. Lepidopterans differ from the non-lepidopteran RyRs at 9 conserved positions: Q^4594^, I^4790^, N^4999^, N^5001^, N^5012^, L^5027^, L^5058^, N^5090^, and T^5141^ (numbering based on *P*. *xylostella* RyR) [37,39,41,115,116]. Four of these (N^4999^, N^5001^, N^5012^, L^5027^) are clustered near the pore-forming segment, while L^5058^ is located in transmembrane helix 6 [37,39,41] and corresponds to I^4862^ in the mouse RyR2, which plays a crucial role in RyR channel activation and gating [117]. Additionally, 8 of the 9 conserved residues (except Q^4594^ corresponding to K^4536^ in DmRyR, GenBank accession number NP_476991) corresponding to M^4748^, D^4957^, K^4959^, H^4970^, I^4985^, I^5016^, G^5048^ and Q^5099^, respectively, in *D. melanogaster* RyR are also conserved amongst non-lepidopteran or invertebrate RyRs [37]. Notably, Q^4594^ is located in the insect divergent region (IDR) with several different amino acids being found at this position, but mostly lysine in Coleoptera, Hymenoptera, and some Diptera [63]. These residues might be involved in differences in channel properties between lepidopteran and non-lepidopteran insect RyRs and in the species with selective toxicity of diamide insecticides [37,41,116]; for further discussion see Section 6. However, the divergence is similar to the two mammalian divergent regions, DR1 and DR2 [118]. The two regions in insect RyRs also exhibit lower similarities to each other and have been defined as insect divergent region 1 (IDR1, amino acids located at 1299–1522 in *L. decemlineata* RyR) and 2 (IDR2, amino acids located at 4395–4721) [41,51,52]. These regions might also be involved in the distinct channel properties of insect RyR isoforms [51]. In contrast, the two EF-hand Ca^2+^ binding motifs originally reported in the lobster RyR [119] are conserved in the carboxy-terminus of insect RyRs (4250–4261 and 4285–4296 in *P. xylostella* RyR) [39]. However, the structural model of PxRyR by Lin et al. [107] revealed that the Ca^2+^ is coordinated by the negatively charged side chains of E^4062^ and E^4136^ in the RIH-associated domain, and the backbone carbonyl of T^5127^ in the C-terminal domain. A relatively recent study on mammalian cardiac RyR2 revealed that the EF-hand domain was not necessary for cytosolic Ca^2+^ activation but required for ER Ca^2+^ [120]. Nevertheless, EF-hand motifs are required for regulation of RyRs by calmodulin [121]. Although this topic requires investigation in insects, binding sites of calmodulin in rabbit RyR1 have already been detected (amino acid positions 3614–3643) [122], and putative corresponding sites have been proposed for insect RyRs (e.g., amino acid positions 3756–3785 in LdRyR) [51].

Insect IP_3_Rs are commonly composed of 2724–2833 residues with a molecular weight of 309–319 kDa (Table 1). No study has examined the crystal structures of insect IP_3_Rs yet. Therefore, the entire structural domain organization and key regions of insect RyRs are based on the predictions of sequence features and comparisons with their mammalian counterparts. Nevertheless, predictions on the structural domain organization of IP_3_Rs reveal differences and are limited to the IP_3_Rs from *D. melanogaster* [61,83], *T. castaneum* [50], and *B. tabaci* [62] (Figure 1). The *D*. *melanogaster* IP_3_R is composed of a middle-coupling domain (N^651^-W^2359^), a putative Ca^2+^-sensor region (G^1986^-S^2354^), and a carboxy-terminal channel-forming domain (S^2360^-Q^2829^) with six transmembrane domains (TM1-TM6) and a pore-forming region [83]. The *B*. *tabaci* IP_3_R contains an inositol 1,4,5-trisphosphate/ryanodine receptor domain (residues 6–229), three MIR domains (residues 116–168, 298–333 and 237–420), two RIH domains (residues 460–664 and 1185–1366), a RIH-associated domain (residues 1918–2037), an oligosaccharide repeat unit polymerase domain (residues 2234–2450), an identity helices domain (residues 4925–5060), and a Sec2p domain (residues 2669–2708) [62]. Troczka et al. [63] conducted a pfam search of conserved domains from insect IP_3_Rs which revealed the presence of six domains, including an IP_3_ binding region, a MIR domain, two RIH domains, a RIH-associated domain, and the transmembrane ion transport domain. The MIR, RIH, RIH-associated regulatory domains at the amino terminus, together with the six transmembrane helices including the GXRXGGGXGD selectivity motif between TM5 and TM6 in the carboxy terminal region, appear to be common to both insect IP_3_Rs and RyRs [50], similar to the mammalian RyRs and IP_3_Rs [91] (Figure 1, Table 2). Notably, there are also functionally orthologous regions, such as the N-terminal regions including the suppressor and ligand binding domains, which lead to modulation of the gating of the Ca^2+^ pore at the carboxy terminus. The 11 residues in the IBC core recognizing IP_3_ in mouse IP_3_R1 [67] are conserved in *T. castaneum* IP_3_R (R^267^, T^268^, T^269^, G^270^, R^271^, R^496^, K^500^, R^503^, Y^560^, R^561^, K^562^) [50]. Additionally, seven residues in the amino-terminal suppression domain of the mouse IP_3_R1 that were shown to be critical for inhibition of IP_3_ binding [74], were also present in TcIP_3_R (L^31^, L^33^, V^34^, D^35^, R^37^, R^55^, K^128^). It is noteworthy that aphid IP_3_Rs appear to create relatively larger channels (around 1000 residues with a molecular weight of 100 kDa) compared to other insect IP_3_Rs (Table 1) [63]. Nevertheless, the overall structural domain organization of *M*. *persicae* IP_3_R does not change other than the additional amino acids scattered across the entire length of the protein, including within the functionally important domains [63]. Larger IP_3_R-like channels are also present in various protozoan species [123,124]. This raises the question whether such divergence is present in other families, which will require identification of more insect IP_3_Rs.

Alternative splicing of RyR mRNA [125,126,127,128] and IP_3_Rs [129] is common in mammalians, leading to differences in Ca^2+^ releasing patterns. The expression of splicing variants of RyRs and IP_3_Rs is regulated both in a tissue-specific and developmental manner. Alternative mRNA splicing was also detected for both insect RyR and IP_3_Rs in many species, with several variants being specific to different tissues and/or developmental stages [33,37,39,41,49,50,51,52,55,56,130], suggesting a functional diversity for RyRs and IP_3_Rs in insect physiology. For example, *B. dorsalis RyR* mRNA possesses four alternative splice variants (ASI-ASIV) [49], while *G*. *molesta* [46], *D. citri* [56], and *T. citricida* [55] RyRs were found to have five, three, and one alternative splicing variant, respectively. Amongst these sites, the splicing site located within the second SPRY domain in the N-terminal part of the channel (amino acids 1135–1167 of the *M*. *persicae* RyR) appears to be quite common in insects [37,40,52,54]. As the second SPRY domain is considered to be a protein–protein interaction domain involved in various biological functions [95,131], splicing variants generated at this location might have different protein–protein interactions [37,63]. *Toxoptera citricida* RyR alternate splicing has been shown to occur by intron retention, a rare splicing event in animals [55]. In contrast, *M*. *persicae* RyR mRNA lacks an alternative splicing variant [54]. On the other hand, at least one alternative splicing site was detected in *D*. *melanogaster* [91] and *T*. *castaneum* (located between amino acid residues 922–929) [50] RyR mRNA. This alternative splice site is also conserved in the human IP_3_R1 [132]. The functional implications of alternative splicing in insect Rys and IP_3_R mRNA has not been studied and requires further investigation.

**Figure 2:**
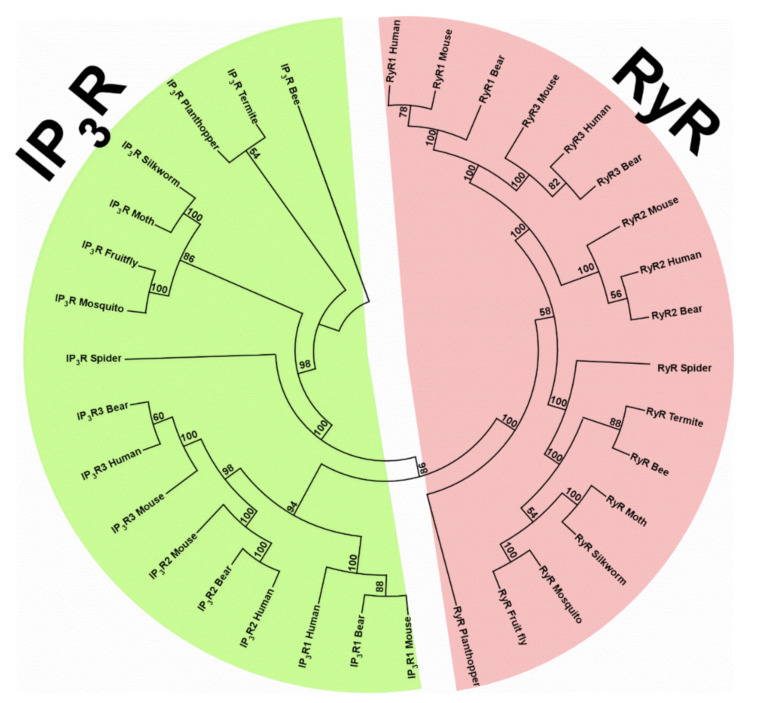
Phylogenetic analysis tree of IP_3_R and RyR, constructed by aligning amino acid sequences from representative species of animal phyla using the MUSCLE algorithm of MEGA-X software, version 10.0 (www.megasoftware.net) (accessed on 21 March 2021) [133]. Phylogenetic trees were constructed by using the maximum likelihood method and Le Gascuel model [134]. The bootstrap consensus tree inferred from 1000 replicates is taken to represent the evolutionary history of the taxa analyzed [135]. Branches corresponding to partitions reproduced in less than 50% bootstrap replicates were collapsed. Representative proteins and their accession numbers are given in Supplementary Table S1.

Phylogenetic analysis of RyRs and IP_3_Rs from a variety of vertebrate and invertebrate species (Table S1) reveals two major clades, the RyR clade and the IP_3_R clade (Figure 2). In each clade, invertebrate and vertebrate RyRs or IP_3_Rs are clustered separately. In invertebrate isoforms of each clade, spider RyR or IP_3_R forms a subclade, while the insect RyRs or IP_3_Rs form another subclade. In the vertebrate isoforms of RyRs, RyR1, and RyR3 isoforms are clustered in one subclade, while RyR2 isoforms are clustered in another subclade. In the vertebrate isoforms of IP_3_Rs, IP_3_R2, and IP_3_R3 isoforms are clustered in one subclade, while IP_3_R1 isoforms are clustered in another subclade. Overall, one could say that each receptor is formed through a gene duplication in invertebrates, which leads to generation of vertebrate RyRs and IP_3_Rs. The three isoforms of each receptor in vertebrates appear to derive via distinct gene duplication events.

## 4. Pathway 

Although RyRs and IP_3_Rs are closely related Ca^2+^ release channels, their regulatory pathways are different [136]. Regardless, reduction in intracellular levels of Ca^2+^ leads to activation of both channels and is primarily coordinated by a process called “Store-Operated Calcium Entry (SOCE)”. Both IP_3_R and RyR are the major biochemical components of the SOCE process and mediate release of Ca^2+^ from the ER into the cytosol or other organelles, such as mitochondria [124,137,138], lysosomes [139,140,141], and the Golgi apparatus [142]. The other major component of this process is the Sarco/endoplasmic reticulum Ca^2+^-ATPase [SERCA], which pumps Ca^2+^ from the cytosol into the ER lumen. There are other players involved in SOCE, for example, the stromal interaction molecule (STIM)-Orai1 complex. STIM is normally located in the ER transmembrane and senses luminal Ca^2+^ depletion, which leads to its translocation to junctions between the ER and plasma membrane where it couples with the plasma membrane Ca^2+^ channel protein Orai1 [143]. This coupling activates Ca^2+^ release-activated Ca^2+^ (CRAC) channels in the plasma membrane, allowing Ca^2+^ influx from the extracellular pools to the cytosol and then from the cytosol to the ER through SERCA [144]. Notably, SERCA might associate with IP_3_R upon depletion of ER Ca^2+^ resulting in enhanced SOCE activity [145,146,147,148]; however, this has not been shown in insect models. Elevation of cytosolic Ca^2+^ to certain levels inactivates CRAC channels thereby terminating Ca^2+^ influx into the cell, a process known as Ca^2+^-dependent inactivation (CDI) [149]. It is noteworthy that the primary Ca^2+^-binding protein, calmodulin, is involved in CDI by binding to STIM, leading to disruption of the STIM-Orai1 complex [150]. The activation of either RyR or IP_3_R is initiated by various external (e.g., light, pheromones, allelochemicals, insecticides) or internal (e.g., neurotransmitters, hormones, growth factors, feeding status, developmental stage, flight) signals that are adjusted based on the biology of insects and associated physiological processes. Activation of the channels might be specific to an organ or cell requiring either the RyR or the IP_3_R.

IP_3_Rs are expressed in most cells, in particular in the ER of neurons [151], fat body adipocytes [Doğan et al., unpublished], and oocytes [152] (Table 2). IP_3_R signaling pathway is integrated with several other signaling pathways, such as the insulin/target of rapamycin (TOR) pathway [153,154]. Low concentrations of cytoplasmic Ca^2+^ activate IP_3_R, while high concentrations (above 300 nM) inhibit channel activity [21,153]. Various receptors in the plasma membrane of the cell, such as G-protein-coupled receptors (GPCRs), stimulate phospholipase C (PLC) that hydrolyzes the phosphorylated plasma membrane glycolipid, phosphatidylinositol 4,5-bisphosphate (PIP_2_), into secondary messengers diacylglycerol (DAG) and IP_3_. IP_3_ binds to IP_3_-binding sites in the N-terminus of the tetrameric IP_3_R to initiate conformational changes that are transmitted down to the transmembrane region leading to opening of the Ca^2+^-permeable pore ~7 nm away from the IBC to release the Ca^2+^ from the ER [155,156]. The IBC form a clam-shaped structure and residues in the IBC required for IP_3_ binding are conserved in IP_3_Rs, but not in RyRs [28,81]. Notably, studies on mammalian IP_3_Rs revealed that IP_3_ binding alone is not sufficient to activate IP_3_Rs [153]. Indeed, IP_3_ binding primes IP_3_Rs to bind Ca^2+^ and Ca^2+^ binding triggers channel opening [157,158]. Insect IP_3_Rs might also require binding of both IP_3_ and Ca^2+^ to open the channel; however, this has not been demonstrated. It is also noteworthy that IP_3_ must bind to each of the four subunits of IP_3_R; the 4- and 5-phosphates of IP_3_ moiety are essential for binding, while the 1-phosphate enhances affinity [159]. Activation of IP_3_R propagates regenerative Ca^2+^ signals by Ca^2+^-induced Ca^2+^ release (CICR) leading to generation of cell-wide Ca^2+^ spikes, oscillations or localized Ca^2+^ “puffs” arising from simultaneous opening of a small cluster of IP_3_Rs [160,161,162]. Calcium spikes through IP_3_R are the main event leading to differential gene expression [153,163]; however, oscillations are also quite common and have been described in many insect cells, including those from salivary glands [164], neurons [165,166], and oocytes [152]. Activity of the IP_3_Rs is also regulated through post-translational modifications, primarily by phosphorylation and dephosphorylation via protein kinases and phosphatases, respectively [167]. For example, the 3′,5′-cyclic monophosphate (cyclic AMP:cAMP)-dependent protein kinase (PKA) phosphorylates IP_3_R resulting in an increase in Ca^2+^ release in mammals [168]. However, *D. melanogaster* IP_3_R lacks PKA sites indicating that it is not regulated by PKA [61]. Other phosphorylation agents, such as the AKT kinase (PKB), protein kinase C (PKC), or Ca^2+^/calmodulin-dependent protein kinase II (CaMKII), might be involved in the phosphorylation of insect IP_3_Rs similar to that in mammalians [83,167,169,170]. IP_3_ is deactivated by phosphorylation to IP_4_ or dephosphorylation to IP_2_ thereby terminating the IP_3_R signaling pathway [171]. Proteins that have EF-hand Ca^2+^-binding motifs, such as calmodulin, can also regulate the activity of the IP_3_Rs. Calmodulin has been shown to inhibit the binding of IP_3_ to IP_3_Rs in mammals in a dose-dependent manner [102,172]. Endogenous calmodulin is essential for the proper activation of the IP_3_R [173]. The direct effect of calmodulin has not been experimentally shown for insect IP_3_Rs; however, in *D. melanogaster*, IP_3_R and calmodulin compete for binding to transient receptor potential (TRP) proteins, which are known to form plasma membrane channels [174]. 

RyRs have a more restricted distribution compared to IP_3_Rs and are predominantly found in the SR of muscle cells and the ER of neurons (Table 2). RyR activation occurs through binding of Ca^2+^ to high affinity binding sites [142,175]. RyR is normally closed at low cytosolic Ca^2+^ (100–200 nM); submicromolar levels of Ca^2+^ act on the RyR channel by increasing open channel probability [92,176,177,178]. A small amount of Ca^2+^ in the cytosol near the receptor causes it to release even more Ca^2+^; however, as the concentration of intracellular Ca^2+^ rises to millimolar concentrations, RyR channel activation becomes inhibited, preventing the total depletion of SR Ca^2+^ [35,179,180,181]. Like cytosolic Ca^2+^, adenine nucleotides also have a biphasic effect on (3H)ryanodine binding [182]; however, this has not been demonstrated for insect RyRs yet. Mammalian RyR activity is regulated by PKA, in particular via the residues in the Repeat34 domain of the channel [69,183]. This phosphorylation has been shown to increase the channel activity [184]. In *P. xylostella* RyR, PKA phosphorylation sites have been detected in the Repeat34 domain, which might regulate the interaction with the neighboring SPRY3 domain [104]. The phosphorylation pattern is temperature-dependent with a lower thermal stability compared to the analogous Repeat34 domain in mammalian RyR isoforms [104]. Notably, mammalian RyR function is known to be modulated also by CaMKII; however, this topic requires investigation in insects (Table 2). On the other hand, the primary Ca^2+^ binding protein, calmodulin has different effects depending on the Ca^2+^ levels and the type of the RyR in mammalians. Calmodulin activates (at low Ca^2+^ levels) or inhibits (at high Ca^2+^ levels) the RyR1 and RyR3 channels, while only inhibitory effects were reported for RyR2 [98,99,185,186]. Although potential calmodulin binding sites have been detected in insect RyRs [33,51], the direct effect of calmodulin on RyR activity in insects has not been demonstrated; however, limited findings provide a hint to calmodulin–channel interaction. *Drosophila melanogaster* calmodulin mutants with a single amino acid change (V91G) were found to possess abnormal Ca^2+^ release in response to depolarization of muscles, which was linked to failed regulation of the RyR [187]. Inhibition of calmodulin has been also shown to enhance the light-induced Ca^2+^ release from internal stores in photoreceptor neurons, indicating calmodulin is involved in the termination of the light response [188,189,190]. Calmodulin rescued the inactivated photoresponse in the presence of ryanodine, suggesting a link between RyR activation and calmodulin action [188,189]. As the activation of the *D*. *melanogaster* visual cascade also includes the cation influx channels transient receptor potential (TRP) protein, which also requires IP_3_R signaling [191], the interaction of calmodulin with both channels in insects requires further investigation.

## 5. Functions

RyRs mediate many cellular and physiological activities, such as muscle contraction, neurotransmitter release, and hormone secretion [17] (Table 2). In accordance with these roles, RyRs are associated with the SR of muscles and the ER of neurons and many other cell types. The mammalian RyR1 and RyR2 are predominately found in skeletal and cardiac muscles, respectively, while RyR3 is relatively abundant in brain and certain skeletal tissues but is also expressed at low levels in multiple tissues [192,193,194]. Neuronal expression of RyR varies, but RyR2 is most abundant. Notably, RYR2 is the major cellular mediator of CICR in animal cells. In contrast to mammalians, there is only one isoform of RyR in insects. The initial studies on insect RyRs have been conducted on *D. melanogaster*. These studies revealed *RyR* is expressed in muscles of the body wall, visceral muscles around the gut, central nervous systems, and optic lobe and retina in the embryonic, larval, and adult stages [32,33,195]. In *D*. *melanogaster* adults, RyR mRNA was detected in tubular muscles and at a lower level in neuronal tissues [32,188] but not ovaries [196,197]. Among head, eyes, antennae and legs, the highest expression was detected in legs [32]. Subsequent studies have examined the site-specific and developmental expression of insect RyR genes in insects other than *D*. *melanogaster*. For example, the highest expression level of *RyR* was detected in the thorax compared with the head and abdomen in adult *B. dorsalis* [49] and *P. rapae* [42], suggesting RyR is involved in the modulation of intracellular Ca^2+^ levels for locomotory activities. Similarly, *RyR* expression was higher in the adult thorax compared to the abdomen; however, the highest expression was detected in the head in *D. citri* [56]. Similar results were also found in *H. armigera* larvae [41], *P. rapae* adults [42], *L. decemlineata* larvae [51], *S. furcifera* nymphs [53] and *T. citricida* adults [55] with higher expression in the head and/or thorax than the abdomen. In contrast, no significant difference in *RyR* expression levels between the head, thorax, and abdomen were detected in the fourth instar larva of *P. xylostella* [39]. A more specific analysis of different tissues in the third instar *L. decemlineata* larvae indicated that *RyR* expression level was highest in foregut, at moderate levels in the hindgut and epidermis, and to a lower extent in the fat body, midgut, ventral ganglia, and Malpighian tubules [51]. In the the fourth instar larvae of *P. rapae*, *RyR* was primarily expressed in the epidermis, at moderate levels in nerve cords, hemocytes, the midgut, and least in the fat body and Malpighian tubules [42]. In the fifth instar larvae of *C. suppressalis*, *RyR* was primarily expressed in the head (including brain and muscle), at moderate levels in the integument and the haemolymph, and least in the fat body, Malpighian tubules, the midgut, and the silk gland [64]. Such distribution of *RyR* mRNAs is not unexpected considering that more muscles are distributed around the foregut, the hindgut, and attached to the epidermis [51]. Nevertheless, the commonly reported higher expression in the thorax and the head are in accordance with the lowest expression in eggs and highest expression in juvenile or adult stages, considering that the mobile stages, such as larvae or adults, require muscle activity. Thus, *RyR* expression was highest in larval or adult stages and lowest in eggs in *O. furnacalis* [40], *B. dorsalis* [49], *H. armigera* [41], *L. decemlineata* [51], and *T*. *castaneum* [50]. Similarly, *RyR* expression was lowest in eggs; however, it was higher in nymphs than adults in *D. citri* [56]. In another hemipteran, *S. furcifera*, *RyR* expression in the fifth instar nymph was significantly higher than in the eggs or female adults; however, no significant difference was detected between the eggs and female adults [53]. This trend is similar to that in *C. suppressalis* with the highest expression in the third instar larvae, but with similar expression in eggs, pupae, and adults [64]. In *N. lugens*, *RyR* transcript levels in female adults were significantly higher than in first to fifth instar nymphs; however, the lowest expression was still in eggs [52]. The expression level of *RyR* in *T. citricida* adults were also found to be significantly higher than those in nymphs [55], while no significant difference in the expression levels of *RyR* was found between nymphs and adults [54]. In contrast to most studies, *RyR* expression levels in eggs, larvae, and adults were all found to be similar in the lepidopteran *P. xylostella* [39]. In brief, these studies, except that by Wang et al. [39], indicate that the expression of *RyR* is higher in adult or juvenile stages (larva or nymph) than in eggs, suggesting involvement of RyRs in locomotory activities. Notably, the immobile pupal stages can also have high expression of *RyR* [40,41,46]. Although most larval muscles are histolyzed during the early-mid phase of pupal development, new muscles are formed at the late pupal stage [198], suggesting that *RyR* expression might fluctuate during pupal transition and be elevated depending on the timing of sampling [51]. It is noteworthy that upregulation of *RyR* expression in pupae might be related to factors other than muscle formation. Notably, *RyR* expression patterns might also be different between sexes. For example, *RyR* expression was found to be significantly higher in males in *S. furcifera* [53], *N. lugens* [52], and *G. molesta* [46]. However, the reason for this sex-dependent variation in insect *RyR* genes is not currently known. Nevertheless, the higher *RyR* expression in the thorax compared to the abdomen is in accordance with the primary function of RyRs in the mediation of excitation-contraction coupling in muscles, which is primarily located in the thorax for mobility [198]. On the other hand, higher expression of *RyR* in the head is in accordance with the involvement of this body part in nerve conduction, hormone secretion and sensory activities, processes that are regulated by RyR activity. It is noteworthy that expression levels of different *RyR* mRNA splicing variants vary between different developmental stages and tissues [33,37,39,40,41,46,49,52,55,65]. In contrast, *M. persicae RyR* mRNA lacks an alternative splicing event, which might be related to its asexual reproduction phase [54]. Alternative splicing of *RyR* mRNAs is common in mammalians with more than 12 distinct splice variants identified to date, leading to important differences in their channel functioning [125,126,199,200]. Some splice variants suppress Ca^2+^ release, while some contribute to distinct Ca^2+^ release patterns [126,127,128]. Interestingly, *T. citricida RyR* mRNA splicing occur by intron retention [55]. Such a splicing event is rare in animals, leading to generation of an optional exon. However, the inclusion of this exon was shown to induce a premature stop codon in *T*. *citricida RyR* mRNA, encoding a truncated protein [55]. Nevertheless, alternative splicing might be critical in generating a diversity of RyRs, leading to subsequent phenotypic changes, in particular for insects which have a single *RyR* gene.

IP_3_Rs are involved in the key events related to the gene expression, development, learning, memory, neuronal signaling, and sensory transduction [129,136] (Table 2). In accordance with these roles, genes encoding IP_3_R are expressed in many cell types, but primarily associated with the ER of neurons. IP_3_R1 is the predominant neuronal isoform and present in endothelial cells, while IP_3_R2 is the predominant isoform in contractile myocardial cells and the sinoatrial node and IP_3_R3 in the intestinal crypt, ovary cells, villus epithelial cells, and the microvillous cells in the olfactory system [201,202,203,204]. Insect genomes possess a single IP_3_R gene. The first *D. melanogaster* IP_3_R gene was reported by Yoshikawa et al. [61] and is expressed mainly in the central nervous system [151], but also other tissues, such as fat body [205] and ovaries [196,197]. A confocal microscopic investigation revealed that IP_3_R is present in all tissues of adult *D*. *melanogaster* and at more homogeneous in levels than RyR [195]. However, the level of transcription in the appendages, containing mainly legs, antennae, wings, and seta, was the highest among all the parts of adult flies [61]. *IP_3_R* mRNA was also abundant in the thorax. Among the head, eyes, antennae and legs, the highest expression was detected at antennae [32]. Developmental expression of *IP_3_R* revealed that the gene is expressed at the highest levels in adults, at moderate levels in eggs, followed by early and mid stage pupa, and least in larvae [61]. Although many studies have been conducted on insect RyRs, the studies on non-Drosophila IP_3_Rs are restricted to only a few insects. Liu et al. [50] reported that the highest and lowest expression levels of *IP_3_R* were detected in 1-day-old larvae and 3-day-old eggs, respectively, in *T. castaneum*. In *B. tabaci*, *IP_3_R* was primarily expressed in larvae, unlike *D. melanogaster*, while expression was moderate in pseudopupa and female adults, and least in eggs [62]. Nevertheless, the higher expression in adults or larvae compared to eggs is similar to those reported for insect *RyR* genes and is in accordance with the possible involvement of IP_3_R in locomotor activities [61], sensory transduction [32] and muscle development [206]. Sex-dependent differential expression of IP_3_R genes was reported from a single insect species. The trend was in favor of females, contrasting to those reported for RyR genes [62]; however, further studies are necessary to make a conclusion. As was reported for *RyR* mRNA, alternative splicing of *IP_3_R* mRNA is also common in mammalians [129]. At least one of these splice sites appears to be conserved in *D. melanogaster* [91].

As we already introduced the site-specific and developmental expression patterns of both RyR and IP_3_R genes, their involvement in insect life processes highlighting lipid metabolism, muscle excitation and contraction in locomotor activities, visualization and olfactory responses, and development are summarized below.

### 5.1. Lipid Metabolism

Various studies in mammals revealed the involvement of Ca^2+^ in lipid metabolism [143,207,208,209,210,211,212,213]. These studies inspired those in insects, which confirmed the involvement of Ca^2+^ in lipid metabolism in insects [214]. The center of the insect lipid metabolism is the fat body, which is primarily composed of the adipocytes that are able to store tremendous amounts of lipids in their cytosolic lipid droplets [214,215,216]. The data on the involvement of Ca^2+^ in insect lipid metabolism is limited and derives mostly from the model insect *D. melanogaster* where increased levels of cytosolic Ca^2+^ in adipocytes lead to fat reduction, whereas decreased cytosolic Ca^2+^ levels induce fat accumulation [217,218,219,220,221,222,223]. Several other studies on non-Drosophila insects also demonstrated the involvement of Ca^2+^ in lipid metabolism, which occurs via the primary Ca^2+^ signaling molecules calmodulin, calcineurin and regucalcin [10]. These studies together indicate that cytosolic Ca^2+^ levels correspond with the levels of triglycerides in lipid droplets. This raises the question as to where RyRs and IP_3_Rs stand in this interaction as the two major intracellular Ca^2+^ suppliers residing in the ER.

Most of the data on the involvement of insect ER Ca^2+^ channels in lipid metabolism are related to IP_3_Rs, which induce lipolysis in insect adipocytes. The loss of *IP_3_R* leads to elevated levels of triglycerides with enlarged lipid droplets in the fat body and hyperphagia in *D*. *melanogaster* adults [218]. In line with this, fat body-specific knockdown of *IP_3_R* leads to an increase in lipid droplet size and triglyceride accumulation in adult flies [222]. The lipolysis is primarily under the control of the adipokinetic hormone (AKH) which binds to AKH-receptor in adipocytes, leading to generation of the secondary messenger cAMP and the PLC [224]. The cAMP induces PKA, leading to activation of the lipolytic transcription factor foxO acting on lipase genes [219]. In parallel, PLC hydrolyzes PIP_2_ to IP_3_, which binds to IP_3_R, leading to activation of the channel and an elevation in cytosolic Ca^2+^ levels [214]. Therefore, AKH activity leads to lipolysis in parallel to the increase in cytosolic levels of Ca^2+^ in adipocytes [214]. While the increase in cytosolic levels of Ca^2+^ transmits the AKH signal, the exact mechanism is not known [219,220,225]. Subramanian et al. [218] reported that reduced insulin signaling in *IP_3_R*-mutants might be one of the reasons for IP_3_R deficiency-related obesity. It is also noteworthy that knockdown of *IP_3_R*, either in all neurons or in peptidergic neurons alone, mimics the *IP_3_R* mutant phenotype with elevated lipid stores and hyperphagia [217]. IP_3_R-mediated Ca^2+^ release in neurons is significantly reduced in these mutants, while the level of short neuropeptide F (sNPF), which is involved in hyperphagia, is elevated [219,220,223] suggesting that IP_3_R-mediated Ca^2+^ signals modulate neural circuits for feeding [218,226,227] and that sNPF is likely to be involved in the activation of IP_3_Rs in neurons [228]. In brief, impaired lipid metabolism derives primarily from peptidergic neurons. These neurons are also associated with the stomatogastric nervous system. On the other hand, AKH-induced lipolysis has been reported only in adults of *D*. *melanogaster* as manipulation of cytosolic Ca^2+^ levels in the larval fat body does not have a significant effect on larval fat stores [219,229]. In contrast, insects, such as *L. decemlineata*, accumulate greater amounts of lipid at the larval stage, which show impaired lipid metabolism upon silencing Ca^2+^-signaling genes [10,216]. Therefore, the dynamics of lipid metabolism in relation to Ca^2+^ might be different depending on the species.

Knowledge on the involvement of RyRs in insect lipid metabolism is restricted to a single study. In *D*. *melanogaster* adults, fat body-specific knockdown of *RyR* leads to an increase in lipid droplet size and triglyceride levels, suggesting a lipolytic role for RyRs [222]. On the other hand, loss of the fat body *seipin* gene in *D*. *melanogaster* adults leads to reduction in triglyceride storage and lipid droplet size, which is linked to impaired SERCA activity, suggesting seipin and SERCA function together to promote fat storage in adipose tissue [222,230]. Interestingly, adipose tissue-specific knockdown of *RyR* partially restores fat storage in *seipin* mutants, while *IP_3_R* silencing did not rescue this phenotype [222]. These findings indicate a complex interaction between the receptors with other molecules involved in Ca^2+^ homeostasis in fat body adipocytes. It is noteworthy that opposite effects were reported on the levels and cellular sites of Ca^2+^ on fat storage in hepatocytes compared to adipocytes in mammals. Increased cytosolic and reduced ER calcium levels induce triglyceride accumulation leading to lipogenesis, whereas reduced cytosolic and increased ER calcium levels reduce triglyceride accumulation leading to lipolysis in hepatocytes and their orthologous cells in the insect fat body, oenocytes [214,222,231]. This suggests that IP_3_R acts as an obesity gene in hepatocytes or oenocytes [222]. However, the data is restricted to *D*. *melanogaster* and, therefore, this topic requires further investigation in other insect species.

### 5.2. Muscle Excitation and Contraction in Locomotor Activities

Calcium is an essential element in the excitation and contraction of muscles [232,233]. ER-released Ca^2+^ is a major source for the stimulation of muscle cells in invertebrates from nematodes towards insects [234,235,236,237]. Insect muscle contraction is similar to that in vertebrate skeletal muscles as in both SR release Ca^2+^ that binds to troponin, a regulatory protein on the thin filament. Troponin activate another regulatory protein, tropomyosin, which causes muscle contraction [238,239]. In contrast, relaxation occurs as the Ca^2+^ pump on the SR membrane transports Ca^2+^ ions back into the SR lumen. This raises the question as to whether RyR or IP_3_R or both are involved in Ca^2+^-related muscle excitation and contraction in insects.

RyRs play a central role in the excitation/contraction (EC) coupling of cardiac and skeletal muscles in mammals [17,240,241]. Studies in *D*. *melanogaster* indicated that *RyR* is mainly expressed in the muscles of the body wall, visceral muscles around the alimentary canal, as well as the central nervous system [33,65,242]. Similarly, high levels of *RyR* expression in muscles have been also reported from non-Drosophila insects, such as *H. virescens* [35] and *L. decemlineata* [51]. Partial loss of *RyR* led to impairment of hypodermal, visceral, and circulatory muscles, indicating RyR is essential for proper muscle function and EC coupling in larval body wall muscles [33,242]. *Drosophila melanogaster RyR* mutants also have a severe defect in the ingestion and passage of food into the gut, confirming that the head and visceral muscles are impaired [242]. On the other hand, mutation calmodulin leads to specific impairment in muscle Ca^2+^ flux, which was found to be related to failed regulation of RyR [187]. RyR activity is also necessary for the spontaneous rhythmic contractions of the lateral oviduct muscles in the cricket, *Gryllus bimaculatus* (Orthoptera: Gryllidae) [237]. Similarly, proctolin induced Ca^2+^ release from the SR, via RyR, plays a major role in hyperneural muscle contractions in *Periplaneta americana* (Blattodea: Blattidae), while IP_3_R-induced Ca^2+^ release has little impact [243].

IP_3_Rs also play a role in the EC and regulation of skeletal, cardiac, and smooth muscle cell functions in mammals [153,244]. Involvement of IP_3_R in insect muscle activity has not been studied in detail. *IP_3_R* is expressed in *D*. *melanogaster* adult muscles, particularly in legs which contain tubular muscles, but to a lesser extent in the thorax, which contains the fibrillary muscles [32,61]. However, it is not known whether IP_3_R has a possible role in tubular or fibrillar muscle function regulation in *D*. *melanogaster*. In *G. bimaculatus*, IP_3_R regulates the amplitude of rhythmic contractions of lateral oviduct muscles; however, the effect was considered minimal [237]. Notably, the inhibitor used in that study, 2-aminoethoxydiphenyl borate, might also inhibit other SOCE molecules, such as SERCA [245], or other volume-regulated anion channels independently from intracellular Ca^2+^ signaling modulation [246]. Further investigation, possibly with other select IP_3_R inhibitors, is required. The involvement of Ca^2+^ in EC of lateral oviduct muscles via the action of several neurohormones was also reported in other studies. For example, octopamine, via the intracellular messenger cAMP, inhibits contraction of the oviducts, while proctolin, via the PLC/IP_3_R, stimulates contraction [247,248,249,250,251]. In *Schistocerca gregaria* (Orthoptera: Acrididae), ryanodine had no effect on proctolin-stimulated foregut muscle contraction, instead, gut muscle contraction was dependent on proctolin receptor-specific activation of the PLC signaling cascade leading to generation of IP_3_ [252]. The authors proposed that the potentiation of contractions by proctolin is mediated by activation of IP_3_-induced Ca^2+^ release from the SR, in contrast to the model of proctolin action on tonic muscle contractions of *P. americana* [243]. These findings support the notion that neurohormones act on the muscles, therefore, their activity is indeed controlled by neuronal signaling pathways [253]. There are various studies on the involvement of neuronal Ca^2+^ levels leading to muscle action, in particular related to locomotor activities such as flight, walking or climbing. For example, the mutations in *IP_3_R* resulted in strong flight deficits in *D*. *melanogaster* [226,254]. Furthermore, pan-neuronal knockdown of the *IP_3_R* leads to significant defects in wing posture in Drosophila, indicating IP_3_R in neurons is necessary during pupal development for flight [227,255]. Examination of Ca^2+^ signals in cultured pupal neurons in *D*. *melanogaster* IP_3_R mutants also revealed high spontaneous Ca^2+^ influx and reduced SOCE, which might lead to loss of flight [256]. These defects and deficits were indeed found to be related to impairment of the IP_3_R signaling induced by neurohormones, primarily the amine-type, and their G-protein coupled receptors in the neurons (e.g., aminergic neurons) [227,254,255,257,258,259]. IP_3_R in neurons can also be induced by other signaling molecules, such as neurotransmitters [256,259], nevertheless, IP_3_R-dependent Ca^2+^ release is essential for neuronal activity. Thus, expression of *IP_3_R* in aminergic neurons during pupal development was found to rescue the adult flight deficit in *D*. *melanogaster IP_3_R* mutants, suggesting the involvement of IP_3_R in flight is related to its role in development [227,254,256]. Other SOCE components, such as STIM-ORAI involved in the extracellular Ca^2+^ influx, are also necessary for normal flight activity [226]. Insect leg muscles are also innervated by neuromodulatory octopaminergic DUM (dorsal unpaired median) neurons or motor neurons [166,260,261,262,263]. In *S. gregaria*, the Ca^2+^ signal in such neurons is dependent on IP_3_R and PLC activation, but not on RyR [264]. In brief, intracellular Ca^2+^ stores in neurons are required for insect rhythmic motor functions which leads to muscle activity and IP_3_R signaling plays a central role in this supply. 

The contradictory results on RyR-induced muscle EC [237,243,265] or IP_3_R- [248,252] still raises questions. The absence of functional genomic studies, such as RNAi, or sophisticated visualization techniques makes it difficult to make conclusive statements on this topic. Nevertheless, the maintenance of intracellular Ca^2+^ levels in muscle cells is a requirement for muscle EC; this probably requires RyR and IP_3_R acting on neuronal pathways.

### 5.3. Visual and Olfactory Sensory Transduction

Visualization and olfactory responses play a crucial role in insect survival as they are involved in accessing food sources, protecting insects from threats, and finding mates to reproduce [266]. This occurs primarily via sensory systems in the eye and antennae; each possesses a small region of tissue, called receptor cells, that are sensitive to a specific stimulus [267,268]. Receptor cells are neurons or other specialized cells and convert odor or light signals into an electrical response that is transmitted to the brain for the processing, a mechanism commonly known as signal transduction [268]. This might be named as “phototransduction” for visualization, and “olfactory sensory transduction” for odor recognition.

Phototransduction starts in ommatidia, units of the insect compound eye that contain sensory neurons known as retinal (visual) cells. The rhabdomere is the central photoreceptive region in each retinal cell and contains photopigment molecules, called rhodopsins [269,270]. Absorption of a photon by rhodopsin leads to activation of the heterotrimeric Gq protein complex, which in turn stimulates PLC to hyrolyzes PIP_2_ to a proton, and the secondary messengers hyrophilic IP_3_ and hyrophobic DAG [267]. The released proton and the mechanical forces caused by PIP_2_ hydrolysis results in opening of light-sensitive, relatively Ca^2+^-selective, “transient receptor potential” (TRP) channels and TRP-like (TRPL) channels which mediate an ionic current responsible for generation of a quantum bump, known as the bump current [271,272,273,274,275]. Calcium is involved in phototransduction; however, studies on the involvement of IP_3_R and RyR are limited. High expression of *IP_3_R* in retina of adult *D*. *melanogaster* suggested a potential role for IP_3_R in visual transduction [32,61]. However, studies on *D*. *melanogaster IP_3_R* mutants revealed that Ca^2+^ release via IP_3_R does not contribute to phototransduction [276,277], instead, PLC activation leads to the opening of light-sensitive Ca^2+^ channels in photoreceptors [278]. A subsequent study in *D*. *melanogaster* proposed that Ca^2+^ release via IP_3_R might have a critical role in light excitation. Silencing of *IP_3_R* specifically in adult photoreceptor cells significantly reduced light-response amplitude in adult photoreceptor cells [279]. Kohn et al. [279] also reported that *IP_3_R* silencing leads to a reduction in PLC catalytic activity, while elevation of intracellular Ca^2+^ rescued the suppressed light responsiveness phenotype. These findings suggest that Ca^2+^ release from internal stores is necessary to increase PLC activity required for bump current, and that functional cooperation between IP_3_R and PLC is necessary for light responsiveness [279]. This study also posits that the reason for lack of connection between *IP3R* and phototransduction in previous studies [276,277] was due to leakage of trace amounts of Ca^2+^ from patchclamp recording electrodes, effectively replacing the Ca^2+^ that would have been released from IP_3_-sensitive stores. However, a more recent study using RNAi or *IP_3_R*-null mutants [280] challenged the work by Kohn et al. [279] supports the the previous findings indicating that IP_3_R does not have a role in phototransduction. Bollepalli et al. [280] argues that phototransduction in *D*. *melanogaster* is compromised by the Gal4 transcription factor used to regulate dsRNA in these experiments, which is not the case for the *IP_3_R* knockdown or mutation in the study by Kohn et al. [279]. These contradictory findings demand further examination on the possible role of IP_3_R in phototransduction. The role of RyR in Ca^2+^ regulation photoreceptor via RyR is equally ambiguous [188,189]. Localization of RyR close to the light-sensitive microvilli in compound eyes of *D*. *melanogaster* suggested a possible role for RyR in Ca^2+^ dependent-phototransduction [281]. However, analysis of mutants in which *RyR* expression was selectively eliminated in the adult eye demonstrated that this channel does not play a role in phototransduction [242]. 

Calcium is also involved in olfactory sensory transduction [282,283,284,285]. Insects perceive odorants with sensory organs called sensilla which are mainly on their antennae. Olfactory sensilla possess tiny pores that project towards olfactory receptor neurons (ORNs) [268]. The dendrites of these bipolar cells extend into a sensillar lumen, while their axons lie in the second (antennal) lobe in the brain. Upon adsorption of an odorant molecule, such as a volatile or an insoluble odorant like a pheromone, in the sensilla, it diffuses into the sensillum via pores, binds to a specific odorant binding protein (OBP) or pheromone binding protein (PBP) in the sensillar lymph and is transferred to olfactory receptors (ORs) on the dendrites of OSNs [286,287,288]. ORs are both ligand-gated and cyclic-nucleotide-activated ion channels and function as heterodimers consisting of a variable odor-specific ligand binding receptor protein that defines their specificity, and a constant highly conserved co-receptor protein, Orco [289,290,291,292]. Orco itself can also act as a non-specific, spontaneously-opening ion channel permeable to Ca^2+^. Other types of receptors are located in different types of sensilla (e.g., ionotropic glutamate-like receptors, gustatory receptors) [268,293,294]. Therefore, both metabotropic and ionotropic signaling mediates odor transduction at ORNs and binding of the odor molecules into ORs leads to cell depolarization and generation of action potentials, which transmit the olfactory signal to the antennal lobe [295]. The transduction mechanism in OSNs is mediated by cAMP relies on PKC instead of PKA, and/or the PLC-linked IP_3_-signaling pathways [290,291,294,296,297,298,299,300,301,302,303,304]. Intracellular Ca^2+^ stores were found to contribute to the ORN responses [285,303,305], raising the question whether IP_3_R and/or RyR are involved in odor transduction pathways. High expression of *IP_3_R* in antennae in adult *D*. *melanogaster* suggests a potential role for IP_3_R in olfactory transduction [32,61]. Additionally, the IP_3_R is present in the olfactory sensory neurons of a variety of species [306,307,308]. However, olfactory responses to a number of different odorants were found to be normal in hypomorphic combinations of *D*. *melanogaster IP_3_R* mutant alleles [257,309]. On the other hand, a subset of these *IP_3_R* alleles, including a null allele, were found to exhibit a faster recovery after a strong odor pulse, suggesting that IP_3_R might be required for maintenance of olfactory adaptation in antennae [309]. In a subsequent study, the magnitude and duration of the odor-induced Ca^2+^ response in ORNs was decreased upon targeting *IP_3_R* and *RyR* by RNAi, as well as by specific blockers, such as thapsigargin or ryanodine [285]. Furthermore, flies expressing *IP_3_R* or *RyR* dsRNA were defective in odor-adaptation [285,303,305]. The magnitude and duration of the Ca^2+^-response was also found to be decreased in cAMP-defective flies based on silencing of the adenyl cyclase gene “*rutabaga*” and the phosphodiesterase gene “*dunce*” [303], in accordance with previous reports that demonstrated involvement of cAMP in olfactory reception [310,311,312]. Furthermore, simultaneous knock-down of *RyR* or *IP_3_R* in combination with knock-down of *rutabaga* and/or *dunce* generated even stronger effects with smaller amplitudes and a shorter duration of Ca^2+^ response to various odors [303]. It is worth noting that when only *IP_3_R* or *RyR* expression is perturbed, perception of odorants (odor-acuity) is not affected, but adaptation to odorants is defective [285]. When cAMP-level is disturbed, odor-perception is affected and the amplitude of the second phase (adaptation to odorants) is completely abolished [303]. Furthermore, in double mutant flies, simultaneous perturbation of both cAMP and IP_3_-signaling severely affects both the first and the second phase and they are unable to detect or adapt to odorants [303]. Therefore, the first phase of olfactory response appears to be mediated by cAMP, which is important for olfactory perception, while the second phase mediated by the intracellular Ca^2+^-signaling pathway is important for odor-adaptation. Due to the limited number of studies, the mechanisms of insect odor transduction are still controversial [298,304,313]. It is also noteworthy that induction of either secondary messenger (cAMP or IP_3_) may be odor-specific [303,311,312,314]. 

In conclusion, evidence as to the role of IP_3_R and RyR in phototransduction or olfactory responses is limited, and further research is required.

### 5.4. Development

Both RyR and IP_3_R have essential roles in development. This is in accordance with the fact that expression of either *RyR* [39,40,41,49,50,51,53,56] or *IP_3_R* [50,62] is up-regulated during development in many insect species. Studies in *D*. *melanogaster* indicated that both genes are also necessary for embryonal development, in particular for development of nervous system and muscles [32,188,189,206].

Loss of *IP_3_R* in *D*. *melanogaster* leads to lethality in the second instar larvae accompanied by delays in molting from the first to the second instar and lower 20-hydroxyecdysone (20E) levels [205,276,315]. A lethal phenotype with a delayed molting is also observed in *PKA* mutants [205,316]. Disruption of either the IP_3_R or cAMP pathway also delays second to third larval instar, third larval instar to pupal, and pupal to adult transitions [205]. Furthermore, *PKA* and *IP_3_R* mutant alleles have a synergistic negative effect on larval molting, suggesting IP_3_R signaling acts in parallel with the cAMP pathway to regulate molting [205]. Exogenous 20E rescues the molting delays caused by disruption of either pathway, suggesting both pathways control 20E levels during molting [205,315]. Indeed, 20E was shown to induce both extracellular and intracellular Ca^2+^ release, leading to activation of PKC and CaMKII that are both involved in 20E-directed gene expression [317,318,319,320]. Similar to that in *D*. *melanogaster*, silencing of *IP_3_R* led to failures in molting and larval-pupal and pupal-adult metamorphosis in the beetle *T. castaneum* [50]. A relatively recent study investigated the larval to pupal switch under nutrient stress in *D*. *melanogaster*, which revealed that the larval-pupal transition requires IP_3_R/Ca^2+^ signaling in glutamatergic interneurons of the mid-ventral ganglion [321]. The nutrient stress sensed by multidendritic cholinergic sensory neurons is conveyed first to glutamatergic interneurons via the acetylcholine receptor, then to medial neurosecretory cells, and finally to the ring gland, leading to stimulation of neuropeptides that induce ecdysteroid biosynthetic genes in the ring gland via IP_3_R signaling to allow pupariation on a protein-deficient diet [321]. The authors suggested that activity in this circuit is an adaptation that provides a layer of regulation to help overcome nutritional stress upon protein deprivation during development. Other studies on neurodevelopment in *D*. *melanogaster* larvae indicated that IP_3_R is essential in particular in aminergic cells for development and survival, and IP_3_R-mediated Ca^2+^ release is required to facilitate release of amine type hormones from aminergic cells or serotonergic and dopaminergic neurons [254,257,258,259,322,323]. Thus, expression of *IP_3_R* in aminergic neurons during pupal development rescues the onset adult flight deficit in *IP_3_R*- *D*. *melanogaster* mutants [227,254]. As *IP_3_R* is also expressed in ovaries in contrast to *RyR* [196,197] and is likely to be involved in Ca^2+^ oscillations in ovaries [152], it may also be necessary for egg activation and ovary development. On the other hand, IP_3_R-mediated Ca^2+^ oscillations also occur in wing imaginal discs that give rise to wings in adults, conferring another role of IP_3_R signaling in development [324]. 

Several studies have examined the role of RyR in insect development. Mutation of *D*. *melanogaster RyR* leads to formation of normal embryos that give rise to larvae with growth defects that die four–seven days during their first instar [242]. Heterozygous individuals containing one copy of the *RyR* mutant allele rescue the calmodulin-lethal phenotypes, further indicating the vital role of RyR [187]. In *T. castaneum*, silencing of *RyR* does not cause any failure in molting or larval-pupal and pupal-adult metamorphosis, in contrast to *IP_3_R* silencing in the same beetle; however, abnormalities in the folding of the hind wings and crawling behavior in adults occur, which might be related to impairment of muscle EC-coupling [50]. 

Developmental physiology also includes topics such as autophagy and the autophagic programmed cell death that play key roles in development, morphogenesis, and regeneration [325,326]. Intracellular Ca^2+^ levels are critical in this respect as lower Ca^2+^ concentrations induce autophagy, while higher Ca^2+^ concentrations switch autophagy to apoptosis [327]. The role of RyR and IP_3_R in these processes is a topic for future investigation.

## 6. Potential of RyR and IP_3_R as Target Sites in Pest Control 

Due to their essential roles, insect Ca^2+^ channels have great potential as target sites for the development of insecticides [328,329,330,331]. As the divergence between mammalian and insect RyRs are greater compared to IP_3_Rs, RyRs might be considered safer targets for insecticidal molecules [332]. While the discovery of diamide insecticides has prompted studies on insect RyRs, no insecticidal compounds targeting IP_3_Rs have been developed to date. The idea of targeting RyRs goes back to the discovery of the plant alkaloid ryanodine from the tropical American shrub, *Ryania speciosa* (Flacourtiaceae), which has high affinity to RyR and interferes with Ca^2+^ signaling in muscles; there receptors are aptly named RyR [333]. Ryanodine keeps the RyR channel partially open leading to Ca^2+^ depletion. The insecticidal activity of *R. speciosa* extracts were first described by Rogers and co-workers in 1946 on a range of lepidopteran and hemipteran pests [334,335]. High toxicity of ryanodine on mammalians was an obstacle to its use as an insecticide; however, it inspired the development of more selective and safer insecticides targeting the operation of RyRs, currently comprised of ryanodine receptor modulators in the Insecticide Resistance Action Committee (IRAC) Group 28 [336]. Based on their common chemistry, these insecticides are generally referred to as diamides.

Diamides are derivatives of benzenedicarboxamide or phthalic acid (flubendiamide, Class I) and anthranilic acid (chlorantraniliprole, cyantraniliprole, and cyclaniliprole, Class II], and selectively activate insect RyRs in the SR and ER in neuromuscular tissues. This causes Ca^2+^ channels to remain partially open leading to an excessive and uncontrollable release of stored Ca^2+^ ions from the ER into the cytosol of muscle cells [337,338,339] resulting in feeding cessation, uncoordinated muscle contraction, paralysis, and death [330,339]. The first diamide registered, flubendiamide, was co-developed by Nihon Nohyaku Co. Ltd. (NNC) and Bayer CropScience [181,332,340,341] and registered in 2006 [340,342]. This was followed by the introduction of chlorantraniliprole [177] developed by DuPont USA in 2007 and cyantraniliprole [343,344] that were co-developed by DuPont and Syngenta in 2008. A fourth chemical, the cyclaniliprole developed by ISK [336], was registered and introduced into the market in 2017, while the most recent one, tetraniliprole developed by Bayer was approved in 2020 [345]. Both benzenedicarboxamide and anthranilic acid derivatives are active against a broad range of lepidopteran pests. The anthranilic acid derivatives are also active sucking hemipterans and coleopterans. Clorantraniliprole has contact, systemic and translaminar activity and exhibits extremely high efficacy against lepidopterans and leaf beetles, as well several dipterans, such as leafminers (*Liriomyza* spp.), isopterans, such as sugar cane termites (*Microtermes obesi*, and *Odontotermes obesus*), and hemipterans, such as whiteflies (*Bemisia* spp.) [343,344,346]. Cyantraniliprole is mainly active against sucking and piercing insects, such as aphids, whiteflies, leafhoppers, psyllids, and thrip due to its systemic properties [344,347,348,349,350]. Cyclaniliprole, is labeled for use against aphids, leaf-feeding caterpillars, mealybugs, thrips, and whiteflies and has contact and translaminar activity [336], while tetraniliprole is labeled for use against white grubs, annual bluegrass weevils, caterpillars, and billbugs (https://www.environmentalscience.bayer.us/turf-and-ornamentals-management/golf-course-management/portfolios-and-solutions/new-bayer-insecticide) (accessed on 4 April 2021).

Diamide insecticides have low mammalian toxicity and are considered safe for beneficial insects and mites, which make them environmentally friendly [343,344]. These features, together with their efficacy, has led to extensive use. A survey on the global insecticide market in 2013 revealed that diamides accounted around 1.2 billion U.S. dollars of global insecticide sales, representing approx. 8% of the insecticide market [336]. The current annual market value is predicted to be around $2.3 billion [351]. This ranks diamides third in the market, accounting for 12% of the global market after neonicotinoids (Group 4A) and synthetic pyrethroids (Group 3A) which account for 24 and 15%, respectively [351]. Additionally, at least three more diamide insecticides (cyhalodiamide, and tetrachlorantraniliprole and unnamed); as well as a third class of diamides, “pyrrole-2 carboxamides” are currently under development, suggesting the use of diamides will continue to increase [345,351,352,353]. However, intensive and repetitive use of the diamides has led to the development of high levels of insecticide resistance in the field, which requires a better understanding of the mode of action of this class of insecticides. 

Diamides act on RyR and induce Ca^2+^ release from intracellular Ca^2+^ stores in insect muscle cells [36,42,338], but also elicit intracellular Ca^2+^ release in isolated insect neurons [177,181,340,354]. Silencing *RyR* in *S. furcifera* [53] or *L. decemlineata* [51] greatly decreases chlorantraniliprole-induced mortality indicating that RyRs are targets of diamides. On the other hand, flubendiamide stimulates SERCA activity, which is attributed to a decrease in ER Ca^2+^ levels [341,355]. Efforts have focused on the binding sites of diamides on RyR. Diamides are incorporated directly into the transmembrane domain of the RyR; however, RyR activation also requires the N-terminus for flubendiamide sensitivity [36]. Deletion experiments on the carboxy-terminal region of the *B*. *mori* RyR revealed that the binding region of flubendiamide is located in the transmembrane domain of the RyR comprising amino acid residues 4111–5084, while the region in the N-terminal cytoplasmic domain correspond to residues at 183–290 [36]. HEK cells expressing either Δ183–290 mutants or a chimeric RyR in which amino acids 4111–5084 were replaced with the counterpart sequence in rabbit RyR2, exhibit failure in Ca^2+^ mobilization in response to flubendiamide, but not to caffeine [36]. A similar study based on the replacement of a 46 amino acid segment (S^4610^-A^4655^) in *D*. *melanogaster* RyR (GenBank accession number: D17389) C-terminal domain with that of a nematode RyR led to insensitivity to diamides [356]. Notably, this shorter region corresponds to A^4659^-A^4703^ in PxRyR, which is within the larger region examined by Kato et al. [36]. However, this region does not overlap with the the highly conserved pore region in *D*. *melanogaster* RyR (aa 4973–4982), where ryanodine binds, or the TM10, which plays a crucial role in human RyR channel activation and gating [97,117,356,357]. A computational modeling approach based on rabbit RyR1 also indicated that I^4790^ and G^4946^ (in *P. xylostella* RyR) are likely to be involved in forming the diamide binding site [358]. On the other hand, radioligand displacement experiments using microsomal membrane preparations of *H. virescens* and *P. americana* muscles indicate that flubendiamide and chlorantraniliprole interact with a binding site that is distinct from the ryanodine binding site [177,178,181,338,359]. Furthermore, radioligand binding studies with house fly muscle membranes provided evidence that flubendiamide and chlorantraniliprole bind at different, allosterically-coupled RyR sites [360]. Recently, a high resolution (3.2 Å) cryo-electron microscopy structure of the rabbit RyR1 in complex with chlorantraniliprole, together with mutagenesis studies revealed that twelve amino acid residues (Y^4697^, K^4700^, Y^4701^, L^4704^, I^4790^, Y^4918^, S^4919^, Y^4922^, D^4942^, V^4943^, G^4946^, and F^4947^ based on *P. xylostella* RyR) comprise the putative chlorantraniliprole binding pocket [361]. Furthermore, a radioligand binding study also suggested that the anthranilic diamides share a common binding site with the pyrrole-2 carboxamides [345]. In brief, despite extensive structural and functional studies, there is not a consensus on the the exact binding site of diamide insecticides. It is also possible that the amino acids in the diamide binding sites of RyRs vary amongst species [56,107,115,116,360,362].

The main goal of identifying diamide binding sites in insect RyRs is related to the development of insecticide resistance and whether there are mutations in these regions that inhibit binding of diamides leading to resistance. Diamide resistance appears to have developed very rapidly as a result of their extensive use due to the lack of alternatives with similar efficacy [363,364]. The initial reports on the development of resistance from field-collected populations have come from *Adoxophyes honmai* (Lepidoptera: Tortricidae) against flubendiamide in Japan [365], *Choristoneura rosaceana* (Lepidoptera: Tortricidae) against chlorantraniliprole in the U.S.A. [366], and *Aphis gossypii* (Hemiptera: Aphididae) against cyantraniliprole in Italy [347], all collected from the field in 2007. This was followed by reports of resistance developed by *P. xylostella* [367], *S. litura* [368], and *S. exigua* against clorantraniliprole in China [369,370], as well as by *B. tabaci* against both clorantraniliprole and cyantraniliprole in the U.S.A. [371], with field collection in 2008 and 2009 for all. In 2010, field-collected samples showed further cases of resistance by *P. xylostella* against flubendiamide and/or clorantraniliprole in Thailand [372] and China [373,374]. In the same year, resistance against clorantraniliprole was found in *A. honmai* in Japan [365] and *C*. *suppressalis* in China [375]. Field populations of at least six lepidopteran species (*P. xylostella*, *C. suppressalis*, *T. absoluta*, *A. honmai*, *S. exigua*, and *S. frugiperda*) and two hemipterans (*A. gossypii* and *B. tabaci*) from 11 countries including Brazil, China, Greece, Italy, Japan, Korea, Mexico, Phillippines, Puerto Rico, Spain, and Thailand have developed moderate to significant levels of resistance (relative ratio ≥10) to diamides (Table 3) [44,47,130,347,358,365,368,369,370,372,373,374,375,376,377,378,379,380,381,382,383,384,385,386,387,388,389,390,391,392,393,394,395,396]. The highest resistance ratios (RRs) 519,157-fold for flubendiamide [387], 288,995-fold for clorantraniliprole [385], 18,423-fold for cyantraniliprole [385], and 11,250-fold for cyclaniliprole [390] (Table 3). The highest resistance levels against flubendiamide were recorded for *P*. *xylostella* populations in Brazil [387] and that against cyclaniliprole for *S. exigua* in Korea [390]. Resistance against clorantraniliprole and cyantraniliprole developed in *T. absoluta* in Brazil [385] (Table 3). On the other hand, lower levels of resistance (Relative Ratio ≤10) have also been reported from various pests, such as *C. medinalis* against chlorantraniliprole [397], *Chrysodeixis includens* against flubendiamide and chlorantraniliprole [398], or by non-lepidopteran species, such as *B. dorsalis* [399] or the aphids *A. gossypii*, and *M. persicae* [347] against cyantraniliprole or whitefly *B. tabaci* against chlorantraniliprole and cyantraniliprole [371]. It is noteworthy that cross-resistance within or between each class of diamides have been also reported [384,400,401,402,403]. This is problematic for new diamides. An investigation on a new diamide, tetraniliprole, in China, which has not been registered yet, revealed that RRs in Chinese field populations of *S. exigua* compared to a susceptible strain were found to be 8.6–128.1, in parallel to the RRs obtained for chlorantraniliprole [394]. This suggests that chlorantraniliprole has cross-resistance with tetraniliprole, as tetraniliprole has not been used in China. Overall, inseciticide resistance management plans should avoid of rotation of anthranilic and phthalic acid diamides [336,404].

Detailed examination of RyRs from field-collected or lab-selected resistant strains revealed mutations that affected residues located in the C-terminal transmembrane spanning domains [358,362,373,376], in accordance with this region being a binding site for diamides. Most of these studies were conducted in *P*. *xylostella*, but to a lesser extent in *T*. *absoluta* and *C. suppressalis*, *S. exigua*, and *S*. *frugiperda*. Four mutations in insect RyRs are associated with diamide resistance; 1) G4946E/V located at the interface between transmemrane domain 4 (TM4) and the TM4-TM5 linker (numbering is based on PxRyR), 2) I4790M/T within the upper TM2 or TM3, 3) E1338D at the N-terminus, and 4) Q4594L in a flexible loop located in DR1 before the pseudo voltage-sensor domain [47,48,107,109,358,362,373,376,378,381,389,393,405]. Ligand binding assays showed that the binding affinity of chlorantraniliprole to native microsomal membranes from field-resistant populations with the G4946E mutation was significantly lower than that in the susceptible strains [358,362]. In another study, binding and efficacy of both flubendiamide and chlorantraniliprole were dramatically impaired in recombinant *P. xylostella* RyR with the G4946E mutation, while affinity to other ligands, such as caffeine or ryanodine, did not change [109]. In a relatively recent study, CRISPR/Cas9 genome-modified *S. exigua* larvae with the G4946E mutation exhibited 223-, 336-, and >1000-fold increase in resistance to chlorantraniliprole, cyantraniliprole and flubendiamide, respectively [402]. Similarly, CRISPR/Cas9 modified *D*. *melanogaster* flies with the G4946V mutation were also found to exhibit high levels of resistance against flubendiamide (RR: 91.3) and chlorantraniliprole (RR:195), but less so against cyantraniliprole (RR:5.4) [405], further indicating the importance of this mutation for diamide resistance. Studies using a recombinant *D*. *melanogaster* RyR with G4946E mutation expressed in Sf9 cells revealed that this mutation confers a high degree of resistance also against pyrrole-2-carboxamides [345]. It is noteworthy that the glycine at position 4946 is conserved amongst insect species, except in the dipteran midge *Belgica antarctica*, the mite *Tetranychus urticae* and the hemipteran mealybug *Ferrisia virgata* [63]. The replacement of glycine with a glutamic acid or valine in the resistant strains is likely to have a major impact on the movement of the S5 and S6 helices, which control opening and closing of the RyR channel pore, leading to an inhibition or decrease in the binding of diamide insecticides to the channel [109,331]. On the other hand, *D*. *melanogaster* flies naturally wild-type for the I4790M mutation exhibit low to moderate resistance to diamides, while the M4790I mutation leads to higher levels of susceptibility to flubendiamide (RR: −15.3 fold), but less to chlorantraniliprole (RR: −7.5) and cyantraniliprole (RR: −2.3) [405]. As mentioned in Section 3. Structure, the isoleucine residue at position 4790 is specific to lepidopterans (in contrast to commonly conserved G^4946^ in insects) as is a methionine in *D*. *melanogaster* and all other insects and arachnids, suggesting I^4790^ might be responsible for the differential sensitivities of the *P. xylostella*, *T. absoluta*, and possibly beetles and other insects to diamides [63,115,116,358,363,373,405]. Homology models of the PxRyR based on rabbit RyR1 indicated that the I4790M mutation in TM2 is located directly opposite to the G4946E mutation (the distance between the two residues is only ~15 Å) in the pseudo voltage sensor domain, suggesting that these two regions might define the diamide binding sites on the receptor [107,109,331,358,362]. The model of PxRyR by Lin et al. [107] further indicated that G4946 is near the entrance to the pocket and that the mutation to glutamatic acid narrows the entrance to the pocket, whereas I4790 is located deep in the pocket and the mutation to methionine makes the pocket shallower. The study by Douris et al. [405] also indicates that G4946V mutations confers very high levels of resistance as the RR of the G4946V mutants to M4790I susceptible mutants is 1400 and 1465 for flubendiamide and chlorantraniliprole, respectively, suggesting both mutations may contribute synergistically to the overall resistance phenotype [406]. Regarding the Q4594L mutation, Q^4594^ is conserved amongst lepidopterans, while I^4790^ is lysine in *D*. *melanogaster* and coleopterans, hymentopterans and some other Dipterans; however, its involvement in diamide binding is not currently known, other than it being mutated in resistant populations [63,373]. The same is valid for E^1338^, which is located in the insect divergent region 2 (IDR2) between SPRY2 and SPRY3 domains and appears not to be conserved in insects [63,107]. A recent study on a Chinese field population of *C. suppressalis* resistant to chlorantraniliprole revealed a new mutation Y4667D/C (corresponding to Y^4701^ in PxRyR), which might confer to high levels of resistance [44]. However, the functional importance of the Y4667D/C, the E1338D and the Q4594L mutations has not been demonstrated to date. 

Other mechanisms might also confer to diamide resistance; this includes metabolic resistance and down-regulation of *RyR*. Metabolic resistance against inseciticides develops through elevated levels of detoxification enzymes, such as cytochrome P450 monooxygenases (P450), glutathione S-transferases (GST) and esterases. The synergistics, piperonyl butoxide (PBO) an inhibitor of P450, diethyl maleate (DEM) a depletor of glutathione, S,S,S-tributylphosphorothioate (DEF) an esterase inhibitor, and triphenyl phosphate (TPP) a carboxylesterase inhibitor, lowered the LC_50_/LD_50_ values of chlorantraniliprole in *L. decemlineata* [407], *P. xylostella* [130], *C. suppressalis* [44] and *S*. *frugiperda* [48]. Additionally, higher levels of cytochrome P450 enzyme and esterases were reported from laboratory strains selected with chlorantraniliprole [44,408,409]. Similarly, transcriptomic profile of chlorantraniliprole-resistant field populations of *P*. *xylostella* revealed that most of the metabolic detoxification enzyme genes were slightly up-regulated [410]. Up-regulation of cytochrome P450 genes by chlorantraniliprole or an increase in the chlorantraniliprole-linked mortality upon silencing of a cytochrome P450 gene have been also reported [411,412,413]. In contrast, synergism tests and biochemical assays showed no obvious correlations between diamide resistance and three detoxifying enzymes in *C*. *suppressalis* [389] and *S*. *exigua* [369]. It is noteworthy indicating that a detoxification mechanism via the ATP-binding cassette (ABC) transporters is also possible [345,414,415]. Down-regulation of *RyR* might also be a possible resistance mechanism to diamide insecticides, which was demonstrated via RNAi in *S. furcifera* [53] and *L. decemlineata* [51]. Down-regulation of *RyR* led to a decrease in the diamide efficacy. In another study, *RyR* was found to be slightly down-regulated in *P*. *xylostella* populations with lower to moderate levels of resistance (RR: 6–35 fold) against chlorantraniliprole, while the gene was significantly down-regulated in a population with high levels of resistance (RR:1750-fold) [410]. Similarly, *RyR* was down-regulated in *C*. *suppressalis* upon treatment with chlorantraniliprole [44]. Down-regulation of *RyR* might slow the release and depletion of intracellular Ca^2+^ stores from the SR in muscles and the ER of many cell types when induced by RyR activators, and consequently enhances resistance to diamide insecticides [53]. It is noteworthy that there are cases reporting over-expression of RyR genes in chlorantraniliprole-resistant populations or up-regulation induced by diamides [38,64,416]. 

As mentioned before, studies on IP_3_Rs as targets in pest control are limited due to their higher similarity with their mammalian counterparts. Nevertheless, a single study has examined the role of IP_3_R in diamide resistance. Interestingly, silencing *IP_3_R* in *B*. *tabaci* adults dramatically decreased susceptibility to cyantraniliprole [62], similar to the decreased chlorantraniliprole-induced mortality upon *RyR* silencing in *S. furcifera* [53] and *L. decemlineata* [51]. It is interesting that continuous administration of cyantraniliprole down-regulates *IP_3_R* expression during the entire period of the treatment in *B*. *tabaci*, which might be a strategy to adjust the RyR-linked increase in intracellular Ca^2+^ and decreased ER Ca^2+^ levels [62]. However, this topic requires further investigation. 

There might be other pest control tools targeting cellular Ca^2+^ homeostasis and interfering with IP_3_R and RyR. Botanicals, entomopathogens, repellents, toxins, Ca^2+^ inhibitors or biomolecules such as dsRNA or miRNAs or peptide agonists or antagonists are promising in this regard. For example, Ma et al. [417] examined the effect of wilforine, a novel botanical insecticide from the root bark of thunder duke vine, *Tripterygium wilfordii* (Celastraceae) [418] on *Mythimna separate* (Lepidoptera: Noctuidae). This investigation revealed that wilforine acts on myocytes leading to an increase in cytosolic Ca^2+^ levels when applied at nanomolar levels and activates both RyR and IP_3_R based on use of specific inhibitors of both channel proteins [417]. Similarly, both IP_3_R and RyR in neurons are activated by the botanical insecticide Celangulin I, extracted from Chinese bittersweet *Celastrus angulatus*, another species from Celastraceae [419]. Other biological agents, such as entomopathogenic viruses, or repellents, such as DEET, or bacterial toxins, such as *Bacillus thuringiensis* Cry toxins might also interfere directly or indirectly with Ca^2+^ signaling and intracellular Ca^2+^ levels [420,421,422,423,424,425,426,427,428,429]. Development of dsRNA-based insecticides interfering with cellular Ca^2+^ homeostasis also has great potential in this manner [10,430,431,432]. Co-application of the agents above with diamides might also have a potential within a combined tactic, which also requires further investigation.

## 7. Conclusions

In conclusion, Ca^2+^ homeostasis is vital for insects, and the ER is one of the major intracellular sources for Ca^2+^. The RyR and IP_3_R are the two channel proteins associated with the ER and are involved in the intracellular Ca^2+^ supply. Insects possess a single RyR and IP_3_R gene, in contrast to mammalians which possess three for each. Both RyR and IP_3_Rs cluster separately in phylogenetic analyses; however, they share common domains, such as the MIR, RIH, RIH-associated regulatory domains at the amino-terminus, and transmembrane helices at the carboxy-terminus. Alternative splicing, which is regulated in a tissue-specific and developmental manner, occurs for both genes and each receptor has its own, distinct, regulatory mechanism. IP_3_R genes are expressed in most cells, in particular in the ER of neurons, adipocytes, and oocytes, while RyR gene expression has a more restricted distribution and is predominantly found in the SR of muscle cells and the ER of neurons. Both receptors have essential roles in insect physiology and development. RyRs mediate many cellular and physiological activities related to muscle contraction and hormone secretion, while IP_3_Rs are involved in key events related to learning, memory, neuronal signaling, lipid metabolism, and sensory transduction. Efforts have concentrated on the development of pest control strategies targeting the operation of RyRs and IP_3_Rs; however, RyRs appear to be safer targets due to their lower similarity with mammalian counterparts compared to IP_3_Rs. Diamides are the best examples of a pest control chemistry targeting RyRs, although resistance developed by pests against diamides has become an increasing issue. Various pest control tactics based on use of botanicals, microbials and toxins, as well as biomolecules such as dsRNA and miRNAs, targeting cellular Ca^2+^ homeostasis and affecting the operation of RyRs and/or IP_3_Rs directly or indirectly might be also promising.

## Figures and Tables

**Table 2:** Comparison of structural and functional features of mammalian and insect RyR and IP_3_Rs.

Receptor	Mammalians							Insects						
	# of Genes	Basic Structure	Primary Exp. Site	Phosphoryl. Status	CaM Binding	Alternative Splicing	Function	# of Genes	Basic Structure	Primary Exp. Site	Phosphory Status	CaM Binding	Alternative Splicing	Function
RyRs	3	N-terminal domain including the A, B, and C subdomains, MIR, RIH, RIHA. SPRY and RyR domains, C-terminal regions with transmembrane domains and EF-hands.	Skeletal and cardiac muscles Central nervous system	PKA CaMKII	+	+	Muscle contraction Neurotransmitter release Hormone secretion	1	N-terminal region including MIR, RIH, three SPRY, RyR repeat, RIHA domains, and a carboxy-terminal region including transmembrane domains and calcium-binding EF-hand domains.	Body wall and visceral muscles Central nervous system and neurons Antenna, eye, and optic lobe Legs Alimentary canal	PKA	Putative binding sites are present.	+	Muscle contraction Locomotor activities Development
IP_3_Rs	3	N-terminal domain including the suppressor (inhibitory) domain (SD) and IP3-binding core β (IBC-β), α (IBC-α) with MIR domain; central modulatory domain including RIH and RIHA domains, C-terminal region with transmembrane domains.	Cerebellum Brain Insulinoma cells Neurons Endothelial, ovary, microvillous and contractile myocardial cells	PKA PKB PKC CaMKII	+	+	Gene expression Development Learning Memory Neuronal signaling Sensory transduction	1	N-terminal domain including MIR domains, a regulatory and transducing region with RIH and RIHA domains, and a carboxy-terminal region including transmembrane domains.	Central nervous system and neurons Fat body adipocytes Ovaries Appendages containing mainly legs, antennae, wings, and seta.	PKA (–) PKB (?) PKC (?) CaMKII (?)	?	+	Locomotor activities Development Visual and olfactory sensory transduction Muscle development Lipid metabolism Hormone secretion

Primary exp. site: primary expression site; phosphoryl. status: phosphorylation status; CaM binding: calmodulin binding.

**Table 3:** Resistance developed by field-populations against diamides to date.

Insecticide	LC_50_ (95%) mg/L or μg/mL	RR^#^	Year	Pest	Population	Country	Reference
**Flubendiamide**	0.16 (0.04–0.8)	1	2009	*Plutella xylostella*	Tub Berg (field susceptible)	Thailand	[372]
	770.8 (123.3–26,336.8)	4817	2011	*Plutella xylostella*	Tha Muang	Thailand	[372]
	10.6 (3.8–22.8)	66	2010	*Plutella xylostella*	Sai Noi	Thailand	[372]
	65.1 (2.7–157.4)	407	2011	*Plutella xylostella*	Sai Noi	Thailand	[372]
	4256.6 (2690.1–9373.2)	26,603	2011	*Plutella xylostella*	Lat Lum Kaew	Thailand	[372]
	0.08 (0.06–0.11)	1	2011	*Plutella xylostella*	Chiang Mai (field susceptible)	Thailand	[376]
	>60	>750	2011	*Plutella xylostella*	Bang Bua Thong	Thailand	[376]
	>200	>1300	2011	*Plutella xylostella*	Sudlon, Cebu Island	Phillippines	[376]
	0.11 (0.08–0.16)	1	2011	*Plutella xylostella*	Roth (lab susceptible)	China	[130]
	1.68 (1.14–2.35)	15	2011	*Plutella xylostella*	Panyu, Guangdong F3	China	[130]
	1.92 (1.19–2.78)	17	2011	*Plutella xylostella*	Zhuhai, Guangdong	China	[130]
	88.5 (66.1–115)	805	2011	*Plutella xylostella*	Zengcheng, Guangdong	China	[130]
	0.9 (0.4–1.4) ***	1	2007	*Plutella xylostella*	Susceptible strain	China	[381]
	22.2 (9.3–35.4) ***	24		*Plutella xylostella*	BY, BaiYun Int. Airport, Guangdong	China	[381]
	1639 (1016–2227) ***	1779		*Plutella xylostella*	ZC, ZengChengi Guangdong	China	[381]
	0.029 (0.026–0.033)	1	2011	*Plutella xylostella*	BCS-S (lab susceptible)	Phillippines	[358]
	>1000	>10,000	2011	*Plutella xylostella*	Sudlon, Cebu Island	Phillippines	[358]
	0.05 (0.03–0.10)	1	2017	*Plutella xylostella*	Susceptible strain	Korea	[390]
	9.6 (2.8–19.4)	192	2017	*Plutella xylostella*	PC, Pyeongchang	Korea	[390]
	1.3 (0.6–2.9)	27	2017	*Plutella xylostella*	GN, Gangneung	Korea	[390]
	0.008 (0.005–0.011)	1	1998	*Plutella xylostella*	RCF-Lab, Recife	Brazil	[387]
	23.0 (7.2–270.1)	2893	2011	*Plutella xylostella*	BNV1, Boas Novas I	Brazil	[387]
	86.1 (23.4–189.7)	1843	2011	*Plutella xylostella*	SPC, Sapucarana	Brazil	[387]
	280.6 (12.9–1038.7)	35,316	2012	*Plutella xylostella*	CGD, Cha Grande	Brazil	[387]
	4111 (2211–8780)	519,157	2012	*Plutella xylostella*	BZR, Bezerros	Brazil	[387]
	0.09 (0.06–0.13)	1	2011/12	*Chilo suppressalis*	Pooled susceptible strains	China	[382]
	1.09 (0.6–2.11)	12	2012	*Chilo suppressalis*	JH12, Jinhua, Zhejiang	China	[389]
	1.08 (0.63–2.11)	12	2013	*Chilo suppressalis*	XS13, Xiangshan, Zhejiang	China	[389]
	1.3 (0.76–2.87)	14	2014	*Chilo suppressalis*	XS14, Xiangshan, Zhejiang	China	[389]
	3.92 (3.02–5.07)	43	2014	*Chilo suppressalis*	YY14, Yuyao, Zhejiang	China	[389]
	0.98 (0.63–1.73)	11	2014	*Chilo suppressalis*	HG14, Huanggang, Hubei	China	[389]
	0.98 (0.64–1.64)	11	2013	*Chilo suppressalis*	SG13, Shanggao, Jiangxi	China	[389]
	0.038 (0.017–0.056)	1	2010	*Tuta absoluta*	GBN, Guaraciaba do Norte-CE	Brazil	[385]
	0.41 (0.34–0.51)	11	2015	*Tuta absoluta*	BZR, Bezerros-PE	Brazil	[385]
	202.8 (153.2–259.9)	5405	2014	*Tuta absoluta*	JDR1 João Dourado I-BA	Brazil	[385]
	221.48 (146.6–312.2)	5901	2014	*Tuta absoluta*	JDR2, João Dourado II-BA	Brazil	[385]
	673.4 (391.3–989.0)	17,943	2014	*Tuta absoluta*	LGD, Lagoa Grande-PE	Brazil	[385]
	1045 (698–1525)	27,854	2014	*Tuta absoluta*	GML2 Gameleira II-BA	Brazil	[385]
	1398 (773–2215)	37,254	2014	*Tuta absoluta*	PSQ Pesqueira-PE	Brazil	[385]
	2178 (1422–3179)	58,044	2014	*Tuta absoluta*	AMD América Dourada-BA	Brazil	[385]
	3018 (2226–3964)	80,413	2014	*Tuta absoluta*	GML1 Gameleira I-BA	Brazil	[385]]
	0.79 (0.3–1.5)	1	2014	*Tuta absoluta*	Lab		[383]
	993 (384–1649)	1257	2014	*Tuta absoluta*	IT-PACH-14-1 Siracusa, Pachino	Italy	[383]
	1376 (792–2772)	1742	2014	*Tuta absoluta*	IT-PACH-14-2 Siracusa, Pachino	Italy	[383]
	1019 (500–2130)	1290	2014	*Tuta absoluta*	IT-GELA-14-1 Caltanissetta, Gela	Italy	[383]
	8.4 (3.6–17.0)	11	2014	*Tuta absoluta*	GR-IER-14-3 Ierapetra, Mpountoules	Greece	[383]
	1.75 (1.36–2.23)	1	2007	*Adoxophyes honmai*	Kanaya (susceptible strain)	Japan	[365]
	55.5 (49.1–63.7)	32	2008	*Adoxophyes honmai*	Shimada-Yui	Japan	[365]
	35.2 (30.1–42.0)	20	2009	*Adoxophyes honmai*	Shimada-Yui	Japan	[365]
	1174 (454 > 10,000)	671	2011 June	*Adoxophyes honmai*	Shimada-Yui	Japan	[365]
	196 (175–221)	112	2011 Aug	*Adoxophyes honmai*	Shimada-Yui	Japan	[365]
	1.54 (1.03–1.97)	1	2007	*Adoxophyes honmai*	Kanaya (susceptible strain)	Japan	[365]
	16.2 (12.9–20.6)	10	2007	*Adoxophyes honmai*	Shimada-Yui	Japan	[365]
	41.8 (37.1–47.2)	27	2008	*Adoxophyes honmai*	Shimada-Yui	Japan	[365]
	24.4 (21.4–28.0)	16	2009	*Adoxophyes honmai*	Shimada-Yui	Japan	[365]
	110 (80.8–173)	71	2010	*Adoxophyes honmai*	Shimada-Yui	Japan	[365]
	141 (119–176)	91	2011 June	*Adoxophyes honmai*	Shimada-Yui	Japan	[365]
	161 (144–181)	105	2011 Aug	*Adoxophyes honmai*	Shimada-Yui	Japan	[365]
	0.001 (0.0002–0.003)	1	2017	*Spodoptera exigua*	Susceptible strain	Korea	[390]
	>100	>100,000	2017	*Spodoptera exigua*	CJ, Cheongju	Korea	[390]
	>100	>100,000	2017	*Spodoptera exigua*	JD, Jindo	Korea	[390]
	9.6 (0.8–27.2)	9560	2017	*Spodoptera exigua*	YG, Yeonggwang	Korea	[390]
	0.66 (0.006–6.51)	660	2017	*Spodoptera exigua*	MR, Miryang	Korea	[390]
	6.5 (5–8.2)	6500	2017	*Spodoptera exigua*	GC, Geochang	Korea	[390]
	0.0007	1		*Spodoptera exigua*	Susceptible strain	Korea	[395]
	0.3 (0.2–0.5)	428	2019	*Spodoptera exigua*	Anseong	Korea	[395]
	10.5 (7.0–14.4)	14,957	2019	*Spodoptera exigua*	Cheongju	Korea	[395]
	210.1 (71.7–295.1)	300,143	2019	*Spodoptera exigua*	Gangneung	Korea	[395]
	52.31 (32.1–70.0)	74,729	2019	*Spodoptera exigua*	Icheon	Korea	[395]
	27.9 (24.1–32.2)	39,929	2019	*Spodoptera exigua*	Jindo	Korea	[395]
	90.4 (67.8–132.0)	129,186	2019	*Spodoptera exigua*	Yeoju	Korea	[395]
	0.003 (0.003–0.005) **	1		*Spodoptera frugiperda*	SUS, Monsanto Company	USA	[391]
	0.03 (0.02–1.5) **	10	2015	*Spodoptera frugiperda*	SIN2015, Sinaloa—Los Mochis	Mexico	[391]
	1.5 (0.8–5.2) **	500	2016	*Spodoptera frugiperda*	PR2016, Ponce	Puerto Rico	[391]
**Clorantraniliprole**	0.225 (0.0535–0.587)	1	2009	*Plutella xylostella*	Tub Berg (field susceptible)	Thailand	[372]
	8 (4.1–13.7)	35	2010	*Plutella xylostella*	Sai Noi	Thailand	[372]
	34.4 (12.1–60.6)	152	2011	*Plutella xylostella*	Sai Noi	Thailand	[372]
	19.7 (7.3–92.4)	87	2011	*Plutella xylostella*	Tha Muang	Thailand	[372]
	174.4 (137.1–219.8)	775	2011	*Plutella xylostella*	Lat Lum Kaew	Thailand	[372]
	0.13 (0.01–0.18)	1	2010	*Plutella xylostella*	Roth (lab susceptible)	China	[374]
	2.4 (1.8–3.7)	18	2010	*Plutella xylostella*	Shenzhen, Guangdong	China	[374]
	10.7 (6.6–26.6)	81	2011	*Plutella xylostella*	Panyu, Guangdong	China	[374]
	265 (184–444)	2000	2011	*Plutella xylostella*	Zengcheng, Guangdong	China	[374]
	18.7 (10.9–28.62)	140	2011	*Plutella xylostella*	Zhuhai, Guangdong	China	[374]
	0.13 (0.09–0.19)	1	2011	*Plutella xylostella*	Roth (lab susceptible)	China	[130]
	2.3 (1.6–3.3)	18	2011	*Plutella xylostella*	Panyu, Guangdong F3	China	[130]
	4 (2.8–5.5)	30	2011	*Plutella xylostella*	Zhuhai, Guangdong	China	[130]
	150 (105–240)	800	2011	*Plutella xylostella*	Zengcheng, Guangdong	China	[130]
	0.30 (0.25–0.38)	1	2011	*Plutella xylostella*	Chiang Mai (field susceptible)	Thailand	[376]
	>60	>200	2011	*Plutella xylostella*	Bang Bua Thong	Thailand	[376]
	>200	>4,100	2011	*Plutella xylostella*	Sudlon, Cebu Island	Phillippines	[376]
	0.007 (0.004–0.012)	1	2011	*Plutella xylostella*	BCS-S (lab susceptible)	Brazil	[380]
	204 (176.9–236.4)	27,793	2011	*Plutella xylostella*	Camocim	Brazil	[380]
	0.006 (0.004–0.008)	1	1998	*Plutella xylostella*	RCF-Lab, Recife	Brazil	[387]
	43.3 (29.7–59.2)	7492	2012	*Plutella xylostella*	BNV2, Boas Novas II	Brazil	[387]
	77.2 (63.6–93.6)	13,365	2012	*Plutella xylostella*	CGD, Cha Grande	Brazil	[387]
	89.6 (75.3–105.9)	15,507	2011	*Plutella xylostella*	SPC, Sapucarana	Brazil	[387]
	112.4 (96.4–130.9)	19,474	2011	*Plutella xylostella*	CSF1, Camocim I	Brazil	[387]
	115.2 (96.3–137.8)	19,944	2011	*Plutella xylostella*	BNV1, Boas Novas I	Brazil	[387]
	123.9 (97–157.3)	21,440	2011	*Plutella xylostella*	JPI, Jupi	Brazil	[387]
	149.1 (113.4–197.7)	25,798	2011	*Plutella xylostella*	CSF2, Camocim II	Brazil	[387]
	162.6 (137.3–193.4)	28,125	2012	*Plutella xylostella*	BZR, Bezerros	Brazil	[387]
	0.011 (0.005–0.018)	1	2010	*Plutella xylostella*	JA (lab susceptible)	Japan	[373]
	23.4 (18.3–31.3)	2128	2010	*Plutella xylostella*	Tonghai city, Yunnan	China	[373]
	0.020 (0.013–0.031)	1	2011	*Plutella xylostella*	BCS-S (lab susceptible)	Phillippines	[358]
	>1000	>10,000		*Plutella xylostella*	Sudlon, Cebu Island	Phillippines	[358]
	0.03 (0.02–0.05)	1	2017	*Plutella xylostella*	Susceptible strain	Korea	[390]
	35.9 (21.1–57.4)	1196	2017	*Plutella xylostella*	PC, Pyeongchang	Korea	[390]
	1.2 (0.4–3)	40	2017	*Plutella xylostella*	GN, Gangneung	Korea	[390]
	0.49 (0.33–0.72)	16	2017	*Plutella xylostella*	SJ, Seongju	Korea	[390]
	0.9 (0.2–1.5) ***	1	2007	*Plutella xylostella*	Susceptible strain	China	[378]
	17.6 (12.5–22.9) ***	20		*Plutella xylostella*	BY, BaiYun Int. Airport, Guangdong	China	[378]
	1954 (1415–2437) ***	2246		*Plutella xylostella*	ZC, ZengChengi Guangdong	China	[378]
	0.82 (0.36–1.5)	1	2011	*Chilo suppressalis*	Fushun11, Fushun, Sichuan (Field Sus.)	China	[375]
	8.4 (5.7–12.2)	10	2010	*Chilo suppressalis*	Yizheng10, Yizheng, Jiangsu	China	[375]
	8.9 (6–14.5)	11	2011	*Chilo suppressalis*	Xiangshan11,Xiangshan, Zhejiang	China	[375]
	10.4 (6.8–15.7)	13	2010	*Chilo suppressalis*	Lujiang10, Lujiang, Anhui	China	[375]
	11.2 (6–20.5)	14	2010	*Chilo suppressalis*	Longyou10, Longyou, Zhejiang	China	[375]
	10.4 (5–23.7)	17	2011	*Chilo suppressalis*	Dong-An11, Dong-An, Hunan	China	[375]
	17.7 (10.6–31.8)	22	2010	*Chilo suppressalis*	Wuxue10, Wuxue, Hubei	China	[375]
	3 (1.4–4.5) ****	1	2012	*Chilo suppressalis*	RA12, Ruian, Zhejiang (Sus. Strain)	China	[379]
	47 (28.4–103) ****	16	2012	*Chilo suppressalis*	ZJ12, Zhuji, Zhejiang	China	[379]
	43.2 (20.1–107.6) ****	14	2013	*Chilo suppressalis*	ZJ13, Zhuji, Zhejiang	China	[379]
	1.4 (1.1–1.7)	1	2011–2012	*Chilo suppressalis*	Pooled susceptible strains	China	[377]
	16.2 (11–27.2)	11	2014	*Chilo suppressalis*	XS14, Xiangshan, Zhejiang	China	[389]
	108.1 (79.5–178.5)	78	2014	*Chilo suppressalis*	YY14, Yuyao, Zhejiang	China	[389]
	0.43 (0.37–0.5)	1	2016	*Chilo suppressalis*	CAAS (lab susceptible)	China	[44]
	108.5 (86.2–136.4)	250	2016	*Chilo suppressalis*	Tong Nan, Nanchang, Jiangxi	China	[44]
	1.4 (1.1–1.7)	1	2011/12	*Chilo suppressalis*	Pooled susceptible strains	China	[377]
	114.5 (71.7–162.1)	82	2016	*Chilo suppressalis*	XS, Xiaoshan, Zhejiang	China	[386]
	199.9 (173.5–229.9)	143	2016	*Chilo suppressalis*	JH, Jinhua, Zhejiang	China	[386]
	147.3 (62.8–280.8)	106	2016	*Chilo suppressalis*	QZ, Quzhou, Zhejiang	China	[386]
	154.8 (103.8–222.1)	111	2016	*Chilo suppressalis*	LY, Longyou, Zhejiang	China	[386]
	195.3 (164.2–232)	140	2016	*Chilo suppressalis*	YQ, Yueqing, Zhejiang	China	[386]
	214 (183.2–250.8)	154	2016	*Chilo suppressalis*	WL, Wenling, Zhejiang	China	[386]
	89.2 (73.9–107)	64	2016	*Chilo suppressalis*	HY, Hengyang, Hu’nan	China	[386]
	109.6 (91.4–131.9)	79	2016	*Chilo suppressalis*	XY, Xinyang, He’nan	China	[386]
	0.18 (0.13–0.30)	1	2014	*Tuta absoluta*	Lab (susceptible strain)		[383]
	47.6 (30.8–77.1)	264	2014	*Tuta absoluta*	IT-PACH-14-1 Siracusa, Pachino	Italy	[383]
	63.7 (42.1–128)	354	2014	*Tuta absoluta*	IT-PACH-14-2 Siracusa, Pachino	Italy	[383]
	435 (165–1193)	2417	2014	*Tuta absoluta*	IT-ACAT-14-1 Ragusa, Acate	Italy	[383]
	225 (135–343)	1250	2014	*Tuta absoluta*	IT-GELA-14-1 Caltanissetta, Gela	Italy	[383]
	2.4 (1.2–17.0)	14	2014	*Tuta absoluta*	GR-IER-14-1 Ierapetra, Kentri	Greece	[383]
	0.0044 (0.0024–0.0068)	1	2014	*Tuta absoluta*	BSL, Brasília-DF	Brazil	[385]
	0.19 (0.12-0.28)	45	2015	*Tuta absoluta*	BZR, Bezerros-PE	Brazil	[385]
	1.5 (1.2–2)	356	2014	*Tuta absoluta*	LGD, Lagoa Grande-PE	Brazil	[385]
	2.3 (1.4–3.4)	525	2014	*Tuta absoluta*	JDR2, João Dourado II-BA	Brazil	[385]
	2.9 (1.9–4.4)	658	2014	*Tuta absoluta*	JDR1 João Dourado I-BA	Brazil	[385]
	4.6 (3.2–7)	1064	2014	*Tuta absoluta*	GML2 Gameleira II-BA	Brazil	[385]
	92.4 (60–129.9)	21,155	2014	*Tuta absoluta*	GML1 Gameleira I-BA	Brazil	[385]
	646 (423–917)	147,928	2014	*Tuta absoluta*	PSQ Pesqueira-PE	Brazil	[385]
	1263 (946–1673)	288,995	2014	*Tuta absoluta*	AMD América Dourada-BA	Brazil	[385]
	0.3 (0.22–0.45)	1	2010	*Tuta absoluta*	Gr-Lab, Peloponnesus	Greece	[388]
	161 (44.2–596)	519	2015	*Tuta absoluta*	GR-IndR, Ierapetra	Greece	[388]
	17 (8.7–42)	55	2015	*Tuta absoluta*	GR-IER-15-2	Greece	[47]
	56 (14–120)	180	2014	*Tuta absoluta*	IT-GELA-14-1, Sicily, Gela	Italy	[47]
	0.21 (0.15–0.29)	1	2005	*Tuta absoluta*	BCS-TA-S, Paulinia, SP	Brazil	[47]
	92 (60–130)	438	2014	*Tuta absoluta*	BR-GML1, Gameleira, BA	Brazil	[47]
	650 (420–920)	3095	2014	*Tuta absoluta*	BR-PSQ, Pesqueira, PE	Brazil	[47]
	1.6 (1.4–1.8)	1	2010	*Adoxophyes honmai*	Kanaya (susceptible strain)	Japan	[365]
	26.3 (21.2–33.8)	17	2010	*Adoxophyes honmai*	Shimada-Yui	Japan	[365]
	64.6 (55.4–78.0)	41	2011 June	*Adoxophyes honmai*	Shimada-Yui	Japan	[365]
	114 (101–132)	73	2011 Aug	*Adoxophyes honmai*	Shimada-Yui	Japan	[365]
	1.3 (1.1–1.5)	1	2010	*Adoxophyes honmai*	Kanaya (susceptible strain)	Japan	[365]
	25.3 (20.7–31.9)	20	2010	*Adoxophyes honmai*	Shimada-Yui	Japan	[365]
	50.0 (43.2–59.0)	39	2011 June	*Adoxophyes honmai*	Shimada-Yui	Japan	[365]
	98.8 (86.7–114)	77	2011 Aug	*Adoxophyes honmai*	Shimada-Yui	Japan	[365]
	0.014 (0.010–0.017)	1		*Spodoptera exigua*	Lab-Sus (susceptible strain)	China	[369]
	0.15 (0.13–0.18)	11	2008	*Spodoptera exigua*	SH08 Minhang, Shanghai	China	[369]
	0.14 (0.11–0.17)	10	2010	*Spodoptera exigua*	SH10 Minhang, Shanghai	China	[369]
	0.14 (0.12–0.16)	10	2008	*Spodoptera exigua*	TA08 Tai’an, Shandong	China	[369]
	0.16 (0.14–0.18)	12	2010	*Spodoptera exigua*	HF10 Hefei, Anhui	China	[369]
	0.21 (0.18–0.25)	15	2010	*Spodoptera exigua*	SZ10 Shengzhen, Guangdong	China	[369]
	0.24 (0.2–0.28)	17	2010	*Spodoptera exigua*	DG10 Dongguang, Guangdong	China	[369]
	0.21 (0.18–0.25)	15	2010	*Spodoptera exigua*	HZ10 Huizhou, Guangdong	China	[369]
	0.16 (0.14–0.19)	12	2010	*Spodoptera exigua*	ZZ10 Zhangzhou, Fujian	China	[369]
	0.37 (0.26–0.52)	1		*Spodoptera exigua*	WH-S (Lab. susceptible)	China	[370]
	12.2 (5.8–35.4)	33	2010	*Spodoptera exigua*	JN, Jinning, Yunnan	China	[370]
	4.7 (2.2–7.9)	13	2009	*Spodoptera exigua*	YL-1, Yanliang, Shanxi	China	[370]
	16.5 (12.6–22)	44	2009	*Spodoptera exigua*	YX, Yongxiu, Jiangxi	China	[370]
	5.3 (1.6–13.9)	14	2009	*Spodoptera exigua*	LG, Longhai, Fujian	China	[370]
	7.5 (3–15.8)	20	2009	*Spodoptera exigua*	HA, Huaian, Jiangsu	China	[370]
	4 (2.6–5.7)	11	2009	*Spodoptera exigua*	LH-1, Luhe, Jiangsu	China	[370]
	3.6 (2.3–6)	10	2010	*Spodoptera exigua*	LH-2, Luhe, Jiangsu	China	[370]
	12.7 (5.1–27.4)	34	2009	*Spodoptera exigua*	FX-1, Fengxian, Shanghai	China	[370]
	6 (3.1–10.8)	16	2010	*Spodoptera exigua*	FX-2, Fengxian, Shanghai	China	[370]
	5.1 (2.4–8.2)	14	2011	*Spodoptera exigua*	FX-3, Fengxian, Shanghai	China	[370]
	0.08 (0.06–0.1)	1		*Spodoptera exigua*	WH-S (Lab. susceptible)	China	[393
	2.2 (1.7–2.9)	27	2014	*Spodoptera exigua*	Baiyun, Guangzhou	China	[396]
	60 (46.1–79.8)	750	2015	*Spodoptera exigua*	Baiyun, Guangzhou	China	[396]
	64 (43.5–87)	800	2016	*Spodoptera exigua*	Baiyun, Guangzhou	China	[396]
	54.5 (41.6–72.3)	682	2017	*Spodoptera exigua*	Baiyun, Guangzhou	China	[396]
	140.7 (106.7–179.1)	1759	2018	*Spodoptera exigua*	Baiyun, Guangzhou	China	[396]
	1.3 (0.97–1.74)	16	2014	*Spodoptera exigua*	Fengxian, Shanghai	China	[396]
	1.9 (1.3–2.6)	24	2015	*Spodoptera exigua*	Fengxian, Shanghai	China	[396]
	45.6 (35–60.7)	571	2016	*Spodoptera exigua*	Fengxian, Shanghai	China	[396]
	159.6 (120.9–210.8)	1995	2017	*Spodoptera exigua*	Fengxian, Shanghai	China	[396]
	207.8 (162.3–267.4)	2597	2018	*Spodoptera exigua*	Fengxian, Shanghai	China	[396]
	0.97 (0.6–1.7)	12	2015	*Spodoptera exigua*	Huangpi, Wuhan	China	[396]
	3.7 (2.6–4.9)	46	2016	*Spodoptera exigua*	Huangpi, Wuhan	China	[396]
	10.3 (7.7–13.6)	129	2017	*Spodoptera exigua*	Huangpi, Wuhan	China	[396]
	17.6 (13.8–22.2)	221	2018	*Spodoptera exigua*	Huangpi, Wuhan	China	[396]
	0.01 (0.0002–0.07)	1	2017	*Spodoptera exigua*	Susceptible strain	Korea	[390]
	>25	>2500	2017	*Spodoptera exigua*	CJ, Cheongju	Korea	[390]
	>25	>2500	2017	*Spodoptera exigua*	JD, Jindo	Korea	[390]
	>25	>2500	2017	*Spodoptera exigua*	YG, Yeonggwang	Korea	[390]
	1.8 (0.8–4.2)	177	2017	*Spodoptera exigua*	MR, Miryang	Korea	[390]
	10.1 (6.5–16.3)	1006	2017	*Spodoptera exigua*	GC, Geochang	Korea	[390]
	0.002	1		*Spodoptera exigua*	Susceptible strain	Korea	[395]
	8 (5.3–12.5)	4000	2019	*Spodoptera exigua*	Anseong	Korea	[395]
	1.2 (0.3–2.7)	600	2019	*Spodoptera exigua*	Cheongju	Korea	[395]
	6.6 (5.3–8.2)	3300	2019	*Spodoptera exigua*	Gangneung	Korea	[395]
	4.6 (2.3–7.0)	2300	2019	*Spodoptera exigua*	Icheon	Korea	[395]
	13.4 (7.6–25.3)	6700	2019	*Spodoptera exigua*	Jindo	Korea	[395]
	21.2 (9.9–498.0)	12,500	2019	*Spodoptera exigua*	Yeoju	Korea	[395]
	0.032 * (0.025–0.041)	1		*Spodoptera exigua*	WH-S strain, Hubei (Susceptible Str.)	China	[393]
	4.9 * (3.9–6.6)	154	2018	*Spodoptera exigua*	WF strain, Weifang, Shandong	China	[393]
	0.055 (0.040–0.072)	1		*Spodoptera exigua*	SS	China	[394]
	9.9 (4.9–19)	180	2018	*Spodoptera exigua*	CL18, Changle, Shandong	China	[394]
	4.1 (1.4–12.4)	74	2019	*Spodoptera exigua*	CL19, Changle, Shandong	China	[394]
	1.5 (1.2–2)	28	2018	*Spodoptera exigua*	AQ18, Anqiu, Shandong	China	[394]
	5.5 (1.8–11.6)	100	2018	*Spodoptera exigua*	NY18, Nanyang, Henan	China	[394]
	4.6 (3.2–6.4)	83	2019	*Spodoptera exigua*	NY19, Nanyang, Henan	China	[394]
	29.3 (17.6–50)	534	2019	*Spodoptera exigua*	AY19, Anyang, Henan	China	[394]
	16.7 (10.6–31.3)	304	2018	*Spodoptera exigua*	XZ18, Xuzhou, Jiangsu	China	[394]
	16.5 (8.7–31.8)	301	2018	*Spodoptera exigua*	XA18, Xian, Shanxi	China	[394]
	136.3 (83.2–229.3)	2477	2019	*Spodoptera exigua*	JX19, Jiaxing, Zhejiang	China	[394]
	4.20 (3.51–4.95)	1		*Spodoptera litura*	XW-Sus (Susceptible Str.)	China	[368]
	47.2 (40.7–53.9)	11	2010	*Spodoptera litura*	SH10, Minhang, Shanghai	China	[368]
	71.6 (54.4–94.9)	17	2008	*Spodoptera litura*	HF08, Hefei, Anhui	China	[368]
	75.5 (61.7–89.8)	18	2010	*Spodoptera litura*	HF10, Hefei, Anhui	China	[368]
	100.3 (84.3–119.3)	24	2009	*Spodoptera litura*	HX09, Hexian, Anhui	China	[368]
	78.9 (64.3–93.5)	19	2010	*Spodoptera litura*	ZZ10, Zhangzhou, Fujian	China	[368]
	102.5 (84–121)	24	2010	*Spodoptera litura*	SZ10, Shenzheng, Guangdong	China	[368]
	80.4 (63.5–96.8)	19	2010	*Spodoptera litura*	HZ10, Huizhou, Guangdong	China	[368]
	98.8 (79.5–118)	23	2010	*Spodoptera litura*	DG10, Dongguang, Guangdong	China	[368]
	0.083 (0.066–0.106)	1		*Spodoptera litura*	SS (Lab. susceptible)	China	[384]
	0.83 (0.65–1.06)	10	2013	*Spodoptera litura*	HZ13, Huizhou, Guangdong	China	[384]
	1.2 (0.9–1.7)	15	2014	*Spodoptera litura*	ZC14, Zengcheng, Guangdong	China	[384]
	0.9 (0.7–1.24)	11	2014	*Spodoptera litura*	ND14, Ningde, Fujian	China	[384]
	1.2 (0.8–1.9)	14	2014	*Spodoptera litura*	HK14, Haikou, Hainan	China	[384]
	1.3 (0.9–1.9)	16	2014	*Spodoptera litura*	GL14, Guilin, Guangxi	China	[384]
	0.001 (0.0007–0.002) **	1		*Spodoptera frugiperda*	SUS, Monsanto Company	USA	[391]
	0.16 (0.06–0.32) **	160	2016	*Spodoptera frugiperda*	PR2016, Ponce	Puerto Rico	[391]
**Cyantraniliprole**	0.0068 (0.0039–0.012)	1	2011	*Plutella xylostella*	BCS-S (susceptible strain)	Phillippines	[358]
	18 (5.1–66)	2647	2011	*Plutella xylostella*	Sudlon, Cebu Island	Phillippines	[358]
	0.009 (0.003–0.03)	1	2017	*Plutella xylostella*	Susceptible strain	Korea	[390]
	0.95 (0.34–2.06)	106	2017	*Plutella xylostella*	PC, Pyeongchang	Korea	[390]
	0.88 (0.35–1.85)	98	2017	*Plutella xylostella*	GN, Gangneung	Korea	[390]
	0.43 (0.24–0.65)	48	2017	*Plutella xylostella*	SJ, Seongju	Korea	[390]
	0.029 (0.025–0.034)	1	1998	*Plutella xylostella*	RCF-Lab, Recife	Brazil	[387]
	0.43 (0.14–0.92)	13	2012	*Plutella xylostella*	BZR, Bezerros	Brazil	[387]
	0.55 (0.25–1.00)	16	2012	*Plutella xylostella*	BNV2, Boas Novas II	Brazil	[387]
	1.3 (0.7–2.2)	39	2011	*Plutella xylostella*	BNV1, Boas Novas I	Brazil	[387]
	10.6 (5.8–18.8)	308	2011	*Plutella xylostella*	SPC, Sapucarana	Brazil	[387]
	33.1 (20.9–56.5)	962	2011	*Plutella xylostella*	CSF2, Camocim II	Brazil	[387]
	37 (31.2–44)	1075	2012	*Plutella xylostella*	CGD, Cha Grande	Brazil	[387]
	64 (43.8–91.9)	1943	2011	*Plutella xylostella*	CSF1, Camocim I	Brazil	[387]
	69.7 (55.4–87.4)	2024	2011	*Plutella xylostella*	JPI, Jupi	Brazil	[387]
	0.08 (0.04–0.13)	1	2017	*Spodoptera exigua*	Susceptible strain	Korea	[390]
	1.8 (1.7–2.2)	23	2017	*Spodoptera exigua*	CJ, Cheongju	Korea	[390]
	>25	>312	2017	*Spodoptera exigua*	JD, Jindo	Korea	[390]
	1.7 (0.01–6.3)	21	2017	*Spodoptera exigua*	YG, Yeonggwang	Korea	[390]
	0.015 (0.011–0.020)	1	2014	*Tuta absoluta*	BSL, Brasília-DF	Brazil	[385]
	1.2 (0.9–1.5)	78	2015	*Tuta absoluta*	BZR, Bezerros-PE	Brazil	[385]
	1.7 (1.2–2.2)	109	2014	*Tuta absoluta*	JDR1 João Dourado I-BA	Brazil	[385]
	2.2 (1.6–3)	147	2014	*Tuta absoluta*	GML2 Gameleira II-BA	Brazil	[385]
	8.5 (6.2–11.4)	556	2014	*Tuta absoluta*	JDR2, João Dourado II-BA	Brazil	[385]
	28.9 (17.3–41.9)	1895	2014	*Tuta absoluta*	LGD, Lagoa Grande-PE	Brazil	[385]
	90.6 (63.3–121.4)	5932	2014	*Tuta absoluta*	GML1 Gameleira I-BA	Brazil	[385]
	152.9 (96.2–224.3)	10,010	2014	*Tuta absoluta*	PSQ Pesqueira-PE	Brazil	[385]
	281.3 (190.8–405)	18,423	2014	*Tuta absoluta*	AMD América Dourada-BA	Brazil	[385]
	0.17 (0.11–0.26)	1		*Aphis gossypii*	171B (Sus. Strain)	Spain	[347]
	2.5 (1.5–3.9)c	14	2010	*Aphis gossypii*	Spain 1, Blanca, Murcia	Spain	[347]
	1.7 (1.4–1.9)	1	2009	*Bemisia tabaci*	MED-S (Sus. Strain)	China	[392]
	43.8 (37.4–51.3)	26	2016	*Bemisia tabaci*	SX, Shanxi	China	[392]
**Cyclaniliprole**	0.002 (0.00009–0.02)	1	2017	*Spodoptera exigua*	Susceptible strain	Korea	[390]
	>22.5	>11,250	2017	*Spodoptera exigua*	CJ, Cheongju	Korea	[390]
	>22.5	>11,250	2017	*Spodoptera exigua*	JD, Jindo	Korea	[390]
	>22.5	>11,250	2017	*Spodoptera exigua*	YG, Yeonggwang	Korea	[390]
	10.7 (4.8–21.2)	5350	2017	*Spodoptera exigua*	MR, Miryang	Korea	[390]
	6.3 (4.9–8.1)	3150	2017	*Spodoptera exigua*	GC, Geochang	Korea	[390]
**Tetraniliprole**	0.04 (0.03–0.07)	1		*Spodoptera exigua*	SS	China	[394]
	1.4 (1–1.9)	33	2018	*Spodoptera exigua*	XA18, Xian, Shanxi	China	[394]
	0.5 (0.3–0.7)	12	2018	*Spodoptera exigua*	NY18, Nanyang, Henan	China	[394]
	5.5 (4.1–7.8)	128	2019	*Spodoptera exigua*	AY19, Anyang, Henan	China	[394]

# LC_50_ of the field populations/LC_50_ of the susceptible strain. Cases with resistance ratios greater than 10-fold are included. * LC_50_ is calculated as μg/cm^2^, LD_50_ values are calculated as μg/μL **, μg/g *** or ng/larva ****. RR stands for resistance ratio. The reference susceptible populations are highlighted.

## Data Availability

Not applicable.

## Supplementary Materials

The following are available online at https://www.mdpi.com/article/10.3390/biom11071031/s1, Table S1: Proteins used in the phylogenetic analysis and alignments in the current review.
