# Supplementary material for: A Comparative Perspective on Functionally-Related, Intracellular Calcium Channels: The Insect Ryanodine and Inositol 1,4,5-Trisphosphate Receptors
Source: Biomolecules. 2021 Jul 15;11(7):1031. doi: 10.3390/biom11071031 (PMC8301844; doi:10.3390/biom11071031)
Supplement: Supplementary file 1 [file biomolecules-11-01031-s001.zip › biomolecules-1292442-supplementary.pdf]

Supplementary Table 1. Proteins used in the phylogenetic analysis and alignments in the current review.

**Ryanodine receptor (RyR)**<br>**Common Name, Species Name (Order: Family) (Protein Accession no)**	**Inositol 1,4,5-trisphosphate receptor (IP_3R)**<br>**Common Name, Species Name (Order: Family) (Protein Accession no)**
RyR1 Human *Homo sapiens* (Primates: Hominidae) (XP_006723380.1)	IP_3R1 Human *Homo sapiens* (Primates: Hominidae) (NP_001093422.2)
RyR2 Human *Homo sapiens* (Primates: Hominidae) (XP_006711865.1)	IP_3R2 Human *Homo sapiens* (Primates: Hominidae) (XP_016874755.1)
RyR3 Human *Homo sapiens* (Primates: Hominidae) (NP_001027.3)	IP_3R3 Human *Homo sapiens* (Primates: Hominidae) (NP_002215.2)
RyR1 Mouse *Mus musculus* (Rodentia: Murida) (XP_036008711.1)	IP_3R1 Mouse *Mus musculus* (Rodentia: Murida) (XP_006505700.1)
RyR2 Mouse *Mus musculus* (Rodentia: Murida) (XP_017170944.1)	IP_3R2 Mouse *Mus musculus* (Rodentia: Murida) (NP_034716.1)
RyR3 Mouse *Mus musculus* (Rodentia: Murida) (XP_017172189.1)	IP_3R3 Mouse *Mus musculus* (Rodentia: Murida) (NP_542120.2)
RyR1 Bear *Ursus arctos horribilis* (Carnivora: Ursidae) (XP_026337550.1)	IP_3R1 Bear *Ursus arctos horribilis* (Carnivora: Ursidae) (XP_026357051.1)
RyR2 Bear *Ursus arctos horribilis* (Carnivora: Ursidae) (XP_026359820.1)	IP_3R2 Bear *Ursus arctos horribilis* (Carnivora: Ursidae) (XP_026358015.1)
RyR3 Bear *Ursus arctos horribilis* (Carnivora: Ursidae) (XP_026335864.1)	IP_3R3 Bear *Ursus arctos horribilis* (Carnivora: Ursidae) (XP_026338459.1)
RyR Bee *Apis mellifera* (Hymenoptera: Apidae) (XP_006569098.1)	IP_3R Bee *Bombus impatiens* (Hymenoptera: Apidae) (XP_024228402.1)
RyR Mosquito *Aedes aegypti* (Diptera: Culicidae) (XP_021707522.1)	IP_3R Mosquito *Aedes aegypti* (Diptera: Culicidae) (XP_021697927.1)
RyR Fruit fly *Drosophila melanogaster* (Diptera: Drosophilidae) (NP_476991.1)	IP_3R Fruit fly *Drosophila melanogaster* (Diptera: Drosophilidae) (NP_001287180.1)
RyR Moth *Spodoptera exigua* (Lepidoptera: Noctuidae) (ALL55467.1)	IP_3R Moth *Manduca sexta* (Lepidoptera: Sphingidae) (XP_037294730.1)
RyR Silkworm *Bombyx mori* (Lepidoptera: Bombycidae) (XP_012544766.2)	IP_3R Sikworm *Bombyx mori* (Lepidoptera: Bombycidae) (XP_037876701.1)
RyR Spider *Stegodyphus dumicola* (Araneae: Eresidae) (XP_035212318.1)	IP_3R Spider *Stegodyphus dumicola* (Araneae: Eresidae) (XP_035209639.1)
RyR Planthopper *Laodelphax striatellus* (Hemiptera: Delphacidae) (AFK84959.1)	IP_3R Planthopper *Nilaparvata lugens* (Hemiptera: Delphacidae) (XP_039297802.1)
RyR Termite *Cryptotermes secundus* (Blattodea: Kalotermitidae) (XP_023711458.1)	IP_3R Termite *Zootermopsis nevadensis* (Isoptera: Archotermopsidae) (XP_021924143.1)
